# A nanoengineered tandem nitroreductase: designing a robust prodrug-activating nanoreactor†

**DOI:** 10.1039/d4cb00127c

**Published:** 2024-11-04

**Authors:** Mariia Zmyslia, Michael J. Capper, Michael Grimmeisen, Kerstin Sartory, Benedikt Deuringer, Mohamed Abdelsalam, Kaiwei Shen, Manfred Jung, Wolfgang Sippl, Hans-Georg Koch, Laurine Kaul, Regine Süss, Jesko Köhnke, Claudia Jessen-Trefzer

**Affiliations:** a Institute of Organic Chemistry, University of Freiburg 79104 Freiburg im Breisgau Germany claudia.jessen-trefzer@pharmazie.uni-freiburg.de; b School of Chemistry, University of Glasgow Glasgow G12 8QQ UK jesko.koehnke@lci.uni-hannover.de; c Institute of Pharmaceutical Science, Pharmaceutical Technology and Biopharmacy, University of Freiburg 79104 Freiburg im Breisgau Germany; d Department of Medicinal Chemistry, Martin-Luther University of Halle-Wittenberg 06120 Halle/Saale Germany; e Department of Pharmaceutical Chemistry, Faculty of Pharmacy, Alexandria University Alexandria Egypt; f Institute for Biochemistry and Molecular Biology, ZBMZ, Faculty of Medicine, University of Freiburg 79104 Freiburg im Breisgau Germany; g Institute of Pharmaceutical Sciences, Chemical Epigenetics Group, University of Freiburg 79104 Freiburg im Breisgau Germany; h CIBSS – Centre for Integrative Biological Signalling Studies, University of Freiburg Schänzlestrasse 18 79104 Freiburg im Breisgau Germany; i Institute of Food Chemistry, Leibniz University Hannover 30167 Hannover Germany

## Abstract

Nitroreductases are important enzymes for a variety of applications, including cancer therapy and bioremediation. They often require encapsulation to improve stability and activity. We focus on genetically encoded encapsulation of nitroreductases within protein capsids, like encapsulins. Our study showcases the encapsulation of nitroreductase NfsB as functional dimers within encapsulins, which enhances protein activity and stability in diverse conditions. Mutations within the pore region are beneficial for activity of the encapsulated enzyme, potentially by increasing diffusion rates. Cryogenic electron microscopy reveals the overall architecture of the encapsulated dimeric NfsB within the nanoreactor environment and identifies multiple pore states in the shell. These findings highlight the potential of encapsulins as versatile tools for enhancing enzyme performance across various fields.

## Abstract 2

Our study demonstrates that encapsulating tandem nitroreductase NfsB within encapsulins, along with pore mutations, enhances enzyme activity, with cryo-EM revealing its structural architecture and multiple pore states.

## Graphical abstract

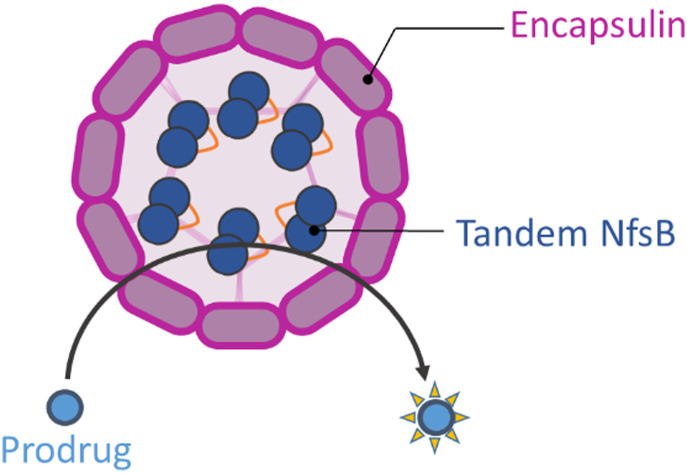

## Introduction

Nitroreductases, versatile enzymes found across various organisms, play pivotal roles in diverse applications spanning bioremediation, cancer therapy, antibiotic production, genetic engineering, biosensors, biocatalysis, and beyond.^1^ These enzymes are typically FMN-depended and catalyze the reduction of nitro aromatic or nitro heterocyclic compounds *via* a ping-pong bi–bi mechanism to yield corresponding hydroxylamines or amines. In the first of two sequential reactions, NADH or NADPH reduces the coenzyme FMN, releasing NAD(P)^+^. In the second reaction, the reduced FMN transfers electrons to the substrate's nitro groups. Historically, *Escherichia coli* (*E. coli*) nitroreductases have been classified into oxygen-insensitive (type I) and oxygen-sensitive (type II) enzymes, which mediate two-electron and single-electron transfers, respectively. NfsB from *E. coli* is a key representative for the type I enzyme class. Although the physiological roles of nitroreductases are still unknown, these enzymes exhibit broad substrate specificity and catalytic activity toward both natural and synthetic compounds, which makes them highly attractive for various applications. In environmental bioremediation, they detoxify nitroaromatic pollutants,^2,3^ while in cancer therapy, they selectively activate prodrugs within tumor cells, or NfsB and NfsA can activate antimicrobials *e.g.* Eeyarestatin 1 (ES1), which is one the rare inhibitors of the bacterial Sec protein channel.^4–12^ Additionally, these enzymes are integral in the biosynthesis of antibiotics and serve as selectable marker in genetic engineering.^13,14^ Nitroreductases also find utility in biosensors for pollutant detection and are employed as biocatalysts for organic synthesis, contributing to pharmaceutical intermediates and fine chemicals.^6,15–17^ Their multifaceted functions underscore their significance in biotechnology, medicine, environmental science, and chemical synthesis. Many applications necessitate the use of an enzyme that remains stable over extended periods, even in challenging environments. Various encapsulation techniques have been developed to protect enzymes, offering potential benefits such as functionalization for purposes like immobilization onto solid supports or targeted delivery to specific tissues.^18–21^ Typically, these techniques involve post-purification modification of the enzymes using structures like liposomes, polysaccharides or organic frameworks. Such manipulations may affect enzyme folding and activity in spite of some encouraging results. An alternative, promising approach involves protein capsids, such as encapsulins, which can be viewed as genetically encoded enzyme encapsulation, and is particularly suitable for immobilization or within physiological conditions.^22–26^ Encapsulins are prokaryotic protein-based nanocompartments. These structures self-assemble into protein shells ranging from 24 to 42 nm in diameter, characterized by the viral HK97-fold of their shell protein. In nature, these nanocompartments can encapsulate specific cargo proteins, such as ferritin-like proteins, peroxidases, and desulfurases. The capsids, comprised of 60–240 subunits arranged in an icosahedral structure, engage with a conserved C-terminal encapsulin localization sequence (ELS), or a more extended N-terminal encapsulation-mediating domain, found on the guest protein. This sequence, also referred to as a targeting peptide (TP) or cargo-loading peptide, effectively guides the guest protein into the interior of the capsid during its self-assembly process.^27,28^ Protein nanocompartments are present across various bacterial and archaeal phyla, and they are believed to be involved in functions such as iron storage, resistance to oxidative stress, anaerobic ammonium oxidation, and sulfur metabolism. Recent genome-mining research has categorized encapsulins into four distinct families (type I–IV), which differ in their sequences, structures, and operon organizations.

Encapsulins are natural protectors against harsh conditions such as proteases, heat, and pH change.^22,23,29–33^ Our recent research has demonstrated the remarkable ability of encapsulins to safeguard chemical catalysts within cellular environments.^34,35^ Moreover, the versatility of encapsulins extends to their outer surface, which can be engineered for diverse applications such as affinity chromatography, immobilization, or cellular targeting.^36–40^ Importantly, engineered encapsulins can be completely genetically encoded and produced in abundance *via* recombinant expression in *E. coli*.

Our study showcases the encapsulation of the nitroreductase NfsB, which we produced as a fused dimer, within encapsulins. Building on our previous studies on the encapsulation of organometallic catalysts, we utilized encapsulin (UniProt ID: I7G8Y9) from the soil organism *Mycobacterium smegmatis* (*M. smegmatis*). This encapsulin belongs to the type I family and exhibits *T* = 1 triangulation symmetry. In our studies, tandem NfsB exhibits enhanced activity and stability across challenging conditions. Using cryogenic electron microscopy (cryo-EM), we extensively characterize the encapsulated nitroreductase and demonstrate its activity against various substrates, which can diffuse through the pores of the capsids to reach NfsB. Furthermore, we show that encapsulated tandem nitroreductase can activate cancer prodrugs in the extracellular environment, suggesting a promising strategy for enzyme-directed prodrug activation in future therapeutic applications.

## Materials and methods

Please see ESI† for details on cloning and procedures, as well as supporting figures and tables.

### Protein production and purification

Corresponding plasmids were transformed into chemically competent *E. coli* BL21 Star (DE3) cells and transformants were selected on LB-agar plates containing ampicillin. A single colony was transferred into LB-medium (supplemented with ampicillin) and grown overnight at 37 °C, 180 rpm. 5 mL pre-culture was transferred into 1 L auto-induction medium (supplemented with ampicillin) and grown at 19 °C for 65 h.

Cells were harvested by centrifugation (5000 × *g*, 4 °C, 30 min) and resuspended at a concentration of 5–6 mL g^−1^ wet weight in lysis buffer (100 mM Tris-HCl, 150 mM NaCl, 1 mM EDTA, pH 8, supplemented freshly before use with 1 mM DTT, 1 mM PMSF and 1 mg mL^−1^ lysozyme; incubation on ice for 30–45 min). Cells were lysed by sonication on ice using a Bandelin Sonopuls HD 2070 ultrasonic homogenizer (Bandelin Electronics, Berlin, Germany, microtip of 3 mm in diameter) for 3 min on/off, 2 times at 40% amplitude. Cell debris was removed *via* centrifugation at 15 000 × *g* for 30 min. Proteins were then purified using 5 mL StrepTrap HP column (Cytiva, Germany) and eluted with elution buffer (100 mM Tris-HCl, 150 mM NaCl, 1 mM EDTA, 2.5 mM desthiobiotin, pH 8). A typical encapsulin purification from 1 L culture yielded approximately 25–30 mg of protein after pooling all fractions from the 5 mL StrepTrap HP column (4.5–5 mg mL^−1^). A typical purification of non-encapsulated cargo proteins NfsB or tandem NfsB (tdNfsB) from 1 L culture yielded approximately 20–25 mg of protein after pooling all fractions from the 5 mL StrepTrap HP column (2.5–3 mg mL^−1^). Further purification was achieved by size exclusion chromatography on a HiLoad 16/600 Superdex 200 PG column (Äkta Protein Purification System, GE Healthcare Germany). Proteins were eluted from the column using an elution buffer. For *in vitro* experiments, proteins were eluted with 50 mM HEPES, 0.05% Tween 20, pH 7.4. For cellular assays, proteins were eluted with 50 mM HEPES, pH 7.4. Following elution, the proteins were reconstituted with a two-fold excess of FMN at 4 °C overnight. The excess unbound FMN was then separated using a PD-10 desalting column with the identical buffer. Fractions were analyzed by SDS-PAGE, pooled, subdivided, and stored at – 80 °C until further use.

### Negative stain-transmission electron microscopy (TEM)

Nanocompartment samples were diluted to an initial concentration of 0.4 mg mL^−1^ in HEPES buffer (50 mM, pH 7.4) and adsorbed for 20 s on carbon-coated grids (Electron Microscopy Sciences, CF300-CU). The grid was stained with 1% uranyl acetate for 20 s. The excess stain was removed using filter paper. TEM images were recorded with a Thermo Scientific Talos L200C transmission electron microscope, operated at 200 kV. The images were analyzed using ImageJ software.

### Dynamic light scattering (DLS)

DLS data were collected on a Zetasizer Nano ZS (Malvern Instruments, Malvern, UK). Measurements were performed at 25 °C using ultra-low volume quartz cuvette (ZEN2112) containing 1 mg mL^−1^ of nanocompartment (Enc{NfsB}, Enc{tdNfsB} or pore mutants) in 50 mM HEPES, 0.05% Tween 20, pH 7.4. Three measurements were performed with the following set up: attenuator: 8; mean count rate (kcps): 260–420. The data were analyzed and presented with Zetasizer Nanoseries software (Malvern Instruments, Malvern, UK) using the general purpose analysis model. The intensity-weighted mean hydrodynamic diameter for each measurement was calculated and recorded.

### SDS-PAGE and gel densitometry

Protein concentrations were determined using the Pierce BCA Protein Assay Kit (Thermo Fisher). Following dilutions were prepared in HEPES buffer (50 mM, pH 7.4): Enc{tdNfsB} at 500 μg mL^−1^; Enc at 90, 225, 360, 495, and 630 μg mL^−1^; and tdNfsB at 26.15, 78.45, 130.75, 183.05, and 235.35 μg mL^−1^. Each protein dilution (20 μL) was mixed with an equal volume (20 μL) of 2× SDS sample buffer (125 mM Tris–HCl (pH 6.8), 4% SDS, 20% (v/v) glycerol, 0.02% bromophenol blue, 200 mM DTT) and then denatured at 95 °C for 10 minutes.

For SDS-PAGE, a 15% gel was prepared in-house and mounted in the SDS-PAGE apparatus. SDS-PAGE running buffer (25 mM Tris, 186 mM Glycine, 0.1% SDS) was added to the bottom and top reservoirs, with the wells washed to remove any trapped air bubbles before sample loading. A volume of 10 μL from each sample was carefully loaded into the wells. The BlueStar Plus Prestained Protein Marker (MWP04, Nippon), which covers a molecular weight range of 10–240 kDa, was included as a standard. The electrodes were connected to a power supply, and electrophoresis was conducted at 200 V and 60 mA for approximately 60 minutes until the bromophenol blue dye front had migrated to the lower edge of the gel.

After electrophoresis, the gels were stained overnight in a solution containing 0.1% Coomassie R-250 in 10% (w/v) ammonium sulfate, 1% (v/v) phosphoric acid, and 10% (v/v) methanol, followed by destaining with water. The destained gels were imaged using the UVP ChemStudio Touch 815 (Analytik Jena) and analyzed with ImageJ software. Background values were subtracted, and calibration curves were plotted for Enc in the range of 45–315 μg per lane and for tdNfsB in the range of 13.08–117.68 μg per lane, correlating the band areas to the respective protein amounts per lane.

The amounts of Enc and tdNfsB in the Enc{tdNfsB} sample were determined using the corresponding calibration curves. The molar concentrations of Enc (1804.2 kDa) and tdNfsB (52.3 kDa) were calculated, and their ratio was assessed to quantify the amount of encapsulated tdNfsB within the Enc nanocompartment. The experiment was repeated twice to ensure reproducibility.

### Blue native PAGE

Blue native PAGE analysis was performed using NativePAGE 3–12% Bis-Tris 1.0 mm Mini Protein Gels (Thermo Fisher) in a XCell Sure-LockTM Mini-Cell gel system (Invitrogen, Darmstadt, Germany). Each sample contained 3 μg of protein and was mixed with 8 μL NativePAGE Sample Buffer (0.5 M 6-aminohexanoic acid, 0.05 g L^−1^ Coomassie Brilliant Blue G250, 37.5% glycerol). Electrophoresis was performed using the following buffer systems: anode buffer (25 mM imidazole/HCl, pH 7.0), dark blue cathode buffer (7.5 mM imidazole/HCl, 50 mM Tricine, 0.2% Coomassie Brilliant Blue G250, pH 7.0) and light blue cathode buffer (7.5 mM imidazole/HCl, 50 mM Tricine, pH 7.0). Electrophoresis started with the dark blue cathode buffer at 120 V for 25 min, followed by a transition to the light blue cathode buffer for an additional 12 hours at 50 V. The voltage was then increased to 120 V for 1 hour, and finally, the gel was subjected to 200 V for the last 30 minutes. After electrophoresis, the gel was stained with Coomassie Brilliant Blue G250 (80 mg dissolved in 1 L of water with 30 mM HCl) and destained with water.

### Grid preparation and data collection

Protein was exchanged into 20 mM HEPES pH 7.5, 100 mM NaCl and concentrated to 20 mg mL^−1^ before application on to Quantifoil 1.2/1.3300 mesh grids. 3 μL were applied to a glow discharged grid in an FEI vitrobot at 8 °C and 95% humidity. Grids were blotted at force 4 for 4 seconds prior to plunge freezing in liquid ethane. Samples were screened on a JEOL F2 microscope prior to extended data collection on a JEOL CRYOARM300 at SCMI (Glasgow, UK). Movies were recorded at a dose of 60.2 e^−^ Å^−2^ using a DE-APOLLO with pixel size 0.7832 Å in super-resolution mode.

### Map reconstruction and model building

Movies were processed in CryoSPARCv.4.3. Briefly, raw movies were motion corrected and 2 × 2 binned using patch motion correction prior to CTF estimation in PatchCTF. Particles were then picked using a low-resolution template produced from screening data on the F2 and resultant particles were extracted 4 × 4 binned in an extraction box size of 512 pixels. The initial particle stack was 2D classified and run through *ab initio* model building with icosahedral symmetry to produce a low-resolution starting model. Particles were subsequently sorted through heterorefinement before re-extraction 2 × 2 binned and further hetero-refinements with “bad” volumes used as sinks to remove poor particles. A final particle stack was reextracted uncropped following reference-based motion correction and run through homogenous refinement with per-particle defocus and CTF-refinement with EWS correction with icosahedral symmetry. The resulting map was sharpened and a model was built *de novo* before refinement and icosahedral expansion in Phenix.

A second map and model were produced by focused local refinement on the pentameric encapsulin around the point of 5-fold symmetry. A mask was produced in ChimeraX to cover the pentameric unit and local refinement was carried out without symmetry restraints. An encapsulin model was then built as a monomer prior to being expanded along map determined icosahedral parameters in Phenix. The resulting model demonstrated a closed pore structure.

Finally, efforts were made to determine the structure of tdNfsB dimers within the encapsulin shell. Particle subtraction was carried out using the final masked volume and 2D classification was used to align the interior components. Density was observed but no high resolution reconstruction could be generated.

### Model building of pore mutants

To predict the structures of the generated encapsulin pore mutants, their amino acid sequences were submitted to the I-TASSER server. I-TASSER employs threading and *ab initio* modeling to generate structure predictions. For alignment, PDB ID 7BOJ, which represents the wild-type encapsulin, was used as the reference template. The model with the highest C-score, reflecting the highest confidence, was chosen for further analysis.

This high-confidence model was then aligned to the pentameric subunit of the 7BOJ template using PyMOL. Surface representations of these pentameric subunits were generated using the APBS plugin in PyMOL to visualize electrostatic potentials. To characterize the structural changes in the five-fold pore, its diameter was measured using MoleOnline (https://mole.upol.cz/).

### Luminescence-based assay for NfsB enzymatic activity

The assay was performed in a white OptiPlate384 microtiter plate (PerkinElmer, Germany) with a total volume of 20 μL per well using an assay buffer containing 50 mM HEPES, 0.05% Tween 20, pH 7.4. The final enzyme concentrations in the reaction mixtures were set to 20 nM NfsB, 10 nM tdNfsB, 3.3 nM Enc{NfsB}, or 3.3 nM Enc{tdNfsB}. For NADH concentration screening, the final reaction mixture contained 25 μM caged-hydroxy-CBT (final DMSO concentration 2.5%), 400 nM FMN, and varying concentrations of NADH at 0, 50 μM, 100 μM, 200 μM, 300 μM, or 400 μM. In the FMN concentration screening, the reaction mixture included 25 μM caged-hydroxy-CBT (final DMSO concentration 2.5%), 400 μM NADH, and varying concentrations of FMN at 0, 50 nM, 100 nM, 200 nM, 300 nM, or 400 nM. For each experimental setup, control wells containing FMN, NADH, and hydroxy-CBT at the same concentrations as the experimental wells were included. All reactions were initiated by adding enzyme dilutions or assay buffer for the negative control wells. The reactions were incubated at 37 °C for 1 hour, after which 20 μL of Luciferin detection reagent (Promega, Germany), supplemented with d-cysteine to a final concentration of 5 mM, was added. After a 20-minute incubation at room temperature, the luminescent signal was measured using a microplate reader (Tecan Spark® Multimode Microplate Reader). All conditions were assessed in triplicate, and the experiment was repeated twice. During the subsequent data evaluation, the readouts from the experimental wells were corrected by subtracting the readouts from the negative control wells before plotting the results.

For the quantification of NfsB enzymatic activity, a luminescence-based assay was conducted using a standard curve of hydroxy-CBT (final DMSO concentration of 2.5%) prepared in the same assay buffer at the following concentrations: 0 μM, 1 μM, 5 μM, 10 μM, 15 μM, 20 μM, and 30 μM. A volume of 20 μL of each standard was pipetted into the wells and incubated at 37 °C for 1 hour. Subsequently, 20 μL of Luciferin detection reagent (Promega, Germany), supplemented with d-cysteine to a final concentration of 5 mM, was added. After a 20-minute incubation at room temperature, the luminescent signal was measured using a microplate reader (Tecan Spark® Multimode Microplate Reader). Each concentration was assessed in triplicate, and the entire experiment was repeated twice. During data evaluation, the readouts from the experimental wells were corrected by subtracting the readouts from the negative control wells (0 μM hydroxy-CBT) before plotting the results.

### *In vitro* prodrug activation

*In vitro* prodrug activation was performed in an assay mixture containing final concentrations: 400 μM NADH, 400 nM FMN, 25 μM of either of the prodrugs (Nbzp,^5^ CB1954,^41^ MA60, MA63, AV2, AV4^4,42^), and either 20 nM tdNfsB, 3.3 nM Enc{tdNfsB} or corresponding pore size mutants. Control samples were prepared with FMN, NADH, and the prodrugs at the same concentrations as in the experimental setup. Each reaction was initiated by adding enzyme dilutions or assay buffer for the negative control setup. The assay was carried out in HEPES buffer (50 mM, pH 7.4) containing 2.5% DMSO as co-solvent at 37 °C at 350 rpm. At designated time points (0.5, 1, 2, 3, 4, 5, 8, 10, 15, 20, 25, 30, 40, 50, 60, 70, 80, 90 minutes for Nbzp; 30 minutes for CB1954, MA60, MA63, AV2, and AV4), aliquots from control and reaction samples were collected and the reactions stopped by mixing 1 : 1 with cold acetonitrile (ACN). The experiment was repeated twice.

Chromatographic separation was achieved on UltiMate 3000 (Thermo Scientific, Germany) using a Polaris 5 C18-A 250 × 4.6 mm column (Agilent, USA). For the analysis of MA60, MA63, CoNO_2_, and CB1954, water was used as mobile phase A, and ACN as mobile phase B. For the analysis of Nbzp, AV2, and AV4 compounds, mobile phase A consisted of water supplemented with 0.1% TFA, and mobile phase B consisted of ACN supplemented with 0.1% TFA. Analysis was performed with a flow rate of 1 mL min^−1^ using the following program: 0–0.5 min at 5% mobile phase B, 0.5–3 min gradient of 5–35% mobile phase B, 3–5 min gradient of 35–95% mobile phase B, 5–8 min at 95% mobile phase B, 8–8.5 min gradient of 95–5% mobile phase B, 8.5–11 min at 5% mobile phase B. The mass spectrometry analysis was performed with an Agilent 6545 LC/Q-TOF system operating in the positive ion mode. During the subsequent quantitative evaluation of prodrug conversion, the area under the curve (AUC) for the prodrug peaks in enzyme-containing samples was divided by the AUC for the corresponding negative controls (without enzyme) and then multiplied by 100% to obtain the percentage conversion.

### Kinetics analysis of nitroreductase activity

The nitroreductase activity of free and encapsulated tdNfsB was assessed by measuring the consumption of NADH at 37 °C. The reactions were performed in HEPES buffer (50 mM, pH 7.4) containing 2.5% DMSO as a co-solvent. The reaction mixtures contained 1.5 mM NADH and variable concentrations of the substrate Nbzp (100, 250, 500, 750, 1000, or 1500 μM). Reactions were initiated by adding the enzyme to achieve final concentrations of 180 nM tdNfsB, 30 nM Enc{tdNfsB}, or corresponding pore size mutants. The assay was conducted in transparent 96-well plates (Sarstedt), with a final reaction volume of 50 μL. Absorbance at 340 nm (A340) was measured in duplicate over a total duration of 30 minutes, with readings taken at 15-second intervals using a Tecan Spark® Multimode Microplate Reader. Background control reactions, lacking protein, were performed and the absorbance values for these controls were subtracted from those of the enzymatic samples to obtain corrected values. The NADH concentration was calculated using the Beer–Lambert law, employing the molar absorption coefficient (extinction coefficient) of NADH at 340 nm (6220 M^−1^ cm^−1^) and a path length of 0.134 cm. Initial reaction rates for the different concentrations of Nbzp were determined by calculating the slope of the linear portion of the initial phase of the time course plots. Subsequently, nonlinear regression analysis was performed on the substrate concentration *versus* initial rate data using the Michaelis–Menten model. Data were plotted in a Lineweaver–Burk plot.

### Assessment of protease susceptibility of free and encapsulated tdNfsB

To evaluate the susceptibility of free and encapsulated tdNfsB to proteolytic degradation, both forms were incubated with Protease from *Streptomyces griseus* (Sigma-Aldrich, P5147). The incubation was conducted using 1 U of protease per 200 pmol of free tdNfsB or per 33.3 pmol of Enc{tdNfsB}, at 37 °C for 10 minutes in 50 mM HEPES, pH 7.4. Following incubation, aliquots were taken and added to the assay mixture to initiate the nitroreductase reaction. The final assay mixture contained 400 μM NADH, 400 nM FMN, and 25 μM CoNO_2_. The final enzyme concentrations in the assay were adjusted to either 20 nM for free tdNfsB or 3.3 nM for Enc{tdNfsB}, with a final volume of 100 μL in a black 96-well plate. Fluorescence measurements were performed after an incubation time of 10 min at 37 °C, using a Tecan Spark 10M plate reader. The excitation wavelength was set to 380 nm, and emission was measured at 510 nm.

### Assessment of thermal stability of free and encapsulated tdNfsB

To evaluate the thermal stability of free and encapsulated tdNfsB, both forms were incubated for 1 min at 55 °C. Following incubation, aliquots were taken and added to the assay mixture to initiate the reaction. The final assay mixture contained 400 μM NADH, 400 nM FMN, and 25 μM CoNO_2_. The final enzyme concentrations in the assay were adjusted to either 20 nM for free tdNfsB or 3.3 nM for Enc{tdNfsB}, with a final volume of 100 μL in a black 96-well plate. Fluorescence measurements were performed after an incubation time of 10 min at 37 °C, using a Tecan Spark 10M plate reader. The excitation wavelength was set to 380 nm, and emission was measured at 510 nm.

### Cell lines and culture conditions

HeLa and H1299 lung carcinoma cell lines were maintained under standard conditions at 37 °C and 5% CO_2_. HeLa cells were cultured in DMEM (high glucose) supplemented with 10% fetal calf serum, while H1299 cells were cultured in RPMI-1640 medium containing 10% fetal calf serum. The cell lines were obtained from the cell line collection at the “Signalling Factory” at University of Freiburg.

### Assessment of activity in HeLa cell lysate of free and encapsulated tdNfsB

A confluent cell culture dish was placed on ice and washed twice with 10 mL of ice-cold PBS. After draining the PBS, 1 mL of ice-cold Pierce IP lysis buffer was added per 150 mm dish. The HeLa cells were scraped off with a cold plastic scraper and transferred into pre-cooled microfuge tubes. The cell suspension was agitated at 4 °C for 30 minutes and then centrifuged at 13 000*g* for 10 minutes at 4 °C. The supernatant was carefully aspirated into fresh pre-cooled tubes and kept on ice, while the pellet was discarded. The protein concentration, as measured with a Nanodrop instrument, was 4–4.4 mg mL^−1^.

The enzyme activity assay was conducted either in Pierce IP lysis buffer or in HeLa cell lysate. The final assay mixture contained 400 μM NADH, 400 nM FMN, and 25 μM CoNO_2_. The enzyme concentrations were adjusted to 20 nM for free tdNTR or 3.3 nM for Enc{tdNfsB}, in a final volume of 100 μL within a black 96-well plate. Fluorescence measurements were performed after an incubation time of 10 min at 37 °C, using a Tecan Spark 10M plate reader, with excitation at 380 nm and emission measured at 510 nm.

### Fluorescence microscopy

For live cell imaging, 300 μL of a 5 × 10^4^ HeLa cell suspension were seeded per well in μ-Slide 8-well chambers with polymer coverslip bottoms (Ibidi) and allowed to attach overnight. After attachment, the medium was replaced with 300 μL of fresh medium containing either 40 μM CoNO_2_, 400 μM NADH, and 400 nM FMN (negative control) or 40 μM CoNO_2_, 400 μM NADH, 400 nM FMN, and 20 nM Enc{tdNfsB}. Cells were incubated for 2 h. Following incubation, the medium was removed and the cells were washed twice with serum-free DMEM. The plasma membrane was stained with CellMask Orange (2.5 μg mL^−1^) prior to imaging (red color).

Fluorescence imaging was performed using an inverted Zeiss LSM 880 laser scanning microscope equipped with an LD LCI Plan-Apochromat 63×/1.40 Oil DIC M27 objective. Green fluorescence (CoNH_2_) was collected between 446–535 nm upon excitation at 405 nm. Red fluorescence (CellMask Orange) was excited at 561 nm and emission was collected between 567–735 nm. All images were acquired under consistent settings to ensure comparability across different experimental conditions.

### Extracellular prodrug activation

H1299 cells were seeded at a density of 2500 cells per well in 96-well plates. After allowing the cells to attach overnight, the medium was removed, and 100 μL of cell culture medium containing 400 μM NADH, 400 nM FMN, and one of the prodrugs (100 μM Nbzp, 100 μM MA60) was added to the wells. Additionally, either 20 nM Enc{eGFP},^34^ Enc{tdNfsB} or 3.33 nM tdNsfB was included in the medium. DMSO (0.5%), 400 μM NADH, 400 nM FMN was used as a negative control. After 72 hours of incubation, the MTS assay was performed as follows: CellTiter 96® AQueous MTS Reagent Powder (Promega, Germany) was prepared as a 1 mg mL^−1^ solution in Dulbecco's phosphate-buffered saline (DPBS), which was then filter-sterilized using a 0.2 μm filter, aliquoted, and stored at −20 °C. Phenazine methosulfate (PMS; Sigma) was prepared at a concentration of 0.92 mg mL^−1^ in DPBS, also filter-sterilized through a 0.2 μm filter, aliquoted, and stored at −20 °C. Immediately before performing the MTS assay, 1.524 mL of the MTS solution was combined with 76 μL of the PMS solution. An additional 8 mL of medium was added to this mixture. In the assay plate, the old medium was replaced with 120 μL of the MTS/PMS solution mixture. Furthermore, 120 μL of the same MTS/PMS mixture in medium was added to empty wells as a negative control. The cells were incubated for 2.5 to 3 hours under standard culture conditions, after which the plate was briefly shaken and measured using a microplate reader (Tecan Spark® Multimode Microplate Reader) at an optical density of 490 nm. All conditions were assessed in triplicate, and the experiment was repeated twice. The average absorbance at 490 nm from the empty “no cell” control wells was substracted from all other absorbance values to obtain corrected absorbance values. The absorbance of untreated cells was set at 100% viability. The viability of experimental wells was calculated by dividing their absorbance by the absorbance of untreated cells and multiplying by 100%.

## Results and discussion

### Construction of the encapsulated nitroreductase enzyme as a single or tandem entity

**Fig. 1:**
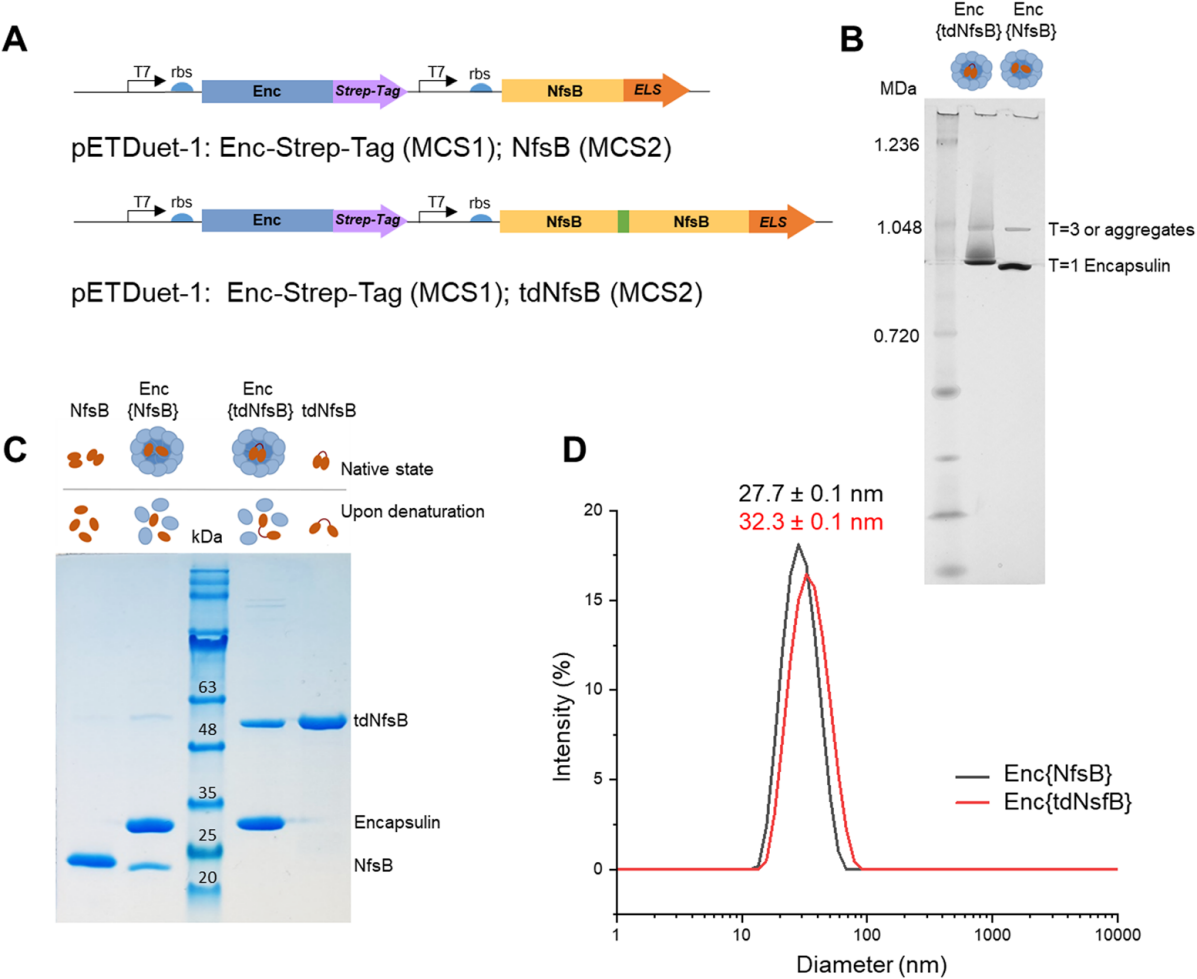
Design and characterization of Enc{NfsB} and Enc{tdNfsB}. (A) Schematic outline of the cloned constructs. (B) Blue Native PAGE analysis of purified Enc{NfsB} and Enc{tdNfsB}. Each sample contained 3 μg of protein per lane in NativePAGE Sample Buffer. (C) SDS-Page analysis of Enc{NfsB} and Enc{tdNfsB}, as well as NfsB and tdNfsB. (D) DLS analysis of Enc{NfsB} and Enc{tdNfsB}.

We inserted the nitroreductase NfsB gene^43^ from *E. coli* into the pETDuet™-1 vector and appended the previously identified C-terminal ELS sequence from *M. smegmatis*^34^ (Ser-Leu-Gly-Ile-Gly-Ser-Leu-Lys-Gly-Thr-Arg). Recognizing that nitroreductase operates as an active dimer, we engineered a tandem enzyme by linking two NfsB copies with a flexible linker sequence (as illustrated in Fig. 1A, for PCR primers see Table S1, ESI†) to ensure obligate dimer formation and thus incorporation into the encapsulin structure.^6^ This decision stemmed from our hypothesis that NfsB monomers would likely be spatially constrained within the capsid, preventing the formation of active dimers once the confined capsid had assembled. The encapsulin gene *msmei_5672 from M. smegmetis mc*^*2*^*155*, tagged with a C-terminal Strep-tag, was cloned under the second T7 promoter in the pETDuet™-1 vector. Following transformation into *E. coli*, we purified the encapsulin particles using affinity chromatography targeting the surface-exposed Strep-tag at the C-terminus of the encapsulin protein. Subsequently, encapsulin was purified by size-exclusion chromatography on a HiLoad 16/600 Superdex 200 PG column (Fig. S1A, ESI†). To further resolve properly formed particles from potential larger aggregates or defective particles we subjected our samples to analysis on a Superose™ 6 Increase 10/300 column, a high-resolution column well suited for investigating large protein assemblies (Fig. S1B, ESI†).^44–46^ This analysis identified a primary product peak at 9.1 mL, with a minor contamination/aggregation peak at 7.5 mL, representing less than 6% of the total area under the curve (AUC) and confirming good sample homogeneity. Control samples, NfsB and tdNfsB without encapsulation, were cloned and purified in accordance with the procedure outlined above (Fig. 1B and Fig. S1C, ESI†).

### Characterization of the purified encapsulated nitroreductase

Blue native-PAGE (BN-PAGE) analysis of the purified constructs unveiled a large protein assembly (Fig. 1B). The upper band likely represents a slightly larger isoform or an aggregate of encapsulin, consistent with observations in other studies and in agreement with the results from our analytical Superose™ 6 runs.^33,47^ Based on the protein standards, the observed bands correspond to approximate sizes of 1.0 and 0.9 MDa, which are lower than the expected molecular weight of the assembled 60-mer (*e.g.* Enc{tdNfsB} ∼2.0 MDa). However, this discrepancy is consistent with findings from other studies, where T1 capsids with a theoretical weight of 1.8 MDa often appear around 1.0 MDa under native-PAGE conditions.^32,47,48^ Under denaturing conditions, distinct protein bands emerged, indicating the presence of encapsulin monomers at the expected size of 30 kDa, and either a tandem nitroreductase (51 kDa), or a single nitroreductase (25 kDa) (Fig. 1C).

Transmission electron microscopy (TEM) and dynamic light scattering (DLS) analyses of the purified encapsulin constructs confirmed the formation of nanocompartments. The average outer diameter measured 23.8 ± 0.5 nm (*n* = 100) for Enc{tdNfsB} and 22.1 ± 1.5 nm (*n* = 100) for Enc{NfsB} (Fig. S1D, ESI†). Moreover, a mean hydrodynamic diameter of 32.3 nm was determined for Enc{tdNfsB}, and 27.7 nm for Enc{NfsB} (Fig. 1D). Notably, both samples exhibited a low polydispersity index (PDI) of 0.10 for Enc{tdNfsB} and 0.08 for Enc{NfsB}, indicating near-monodisperse characteristics.

In summary, our method produces encapsulins of high purity with minimal contamination or aggregation, as indicated by less than 6% impurities detected *via* Superose™ 6 chromatography. The particles are nearly monodisperse, exhibiting a PDI that is comparable to or better than those typically reported for native and engineered encapsulin samples.^44,49^ This improved homogeneity is likely attributable to our purification process, which utilizes a purification tag and does not involve precipitation, but instead employs affinity chromatography. Additionally, the observed outer diameter of ∼24 nm is consistent with the reported diameters of T1 capsids observed by us and other research groups.

### Overall structure of Enc{tdNfsB}

**Fig. 2:**
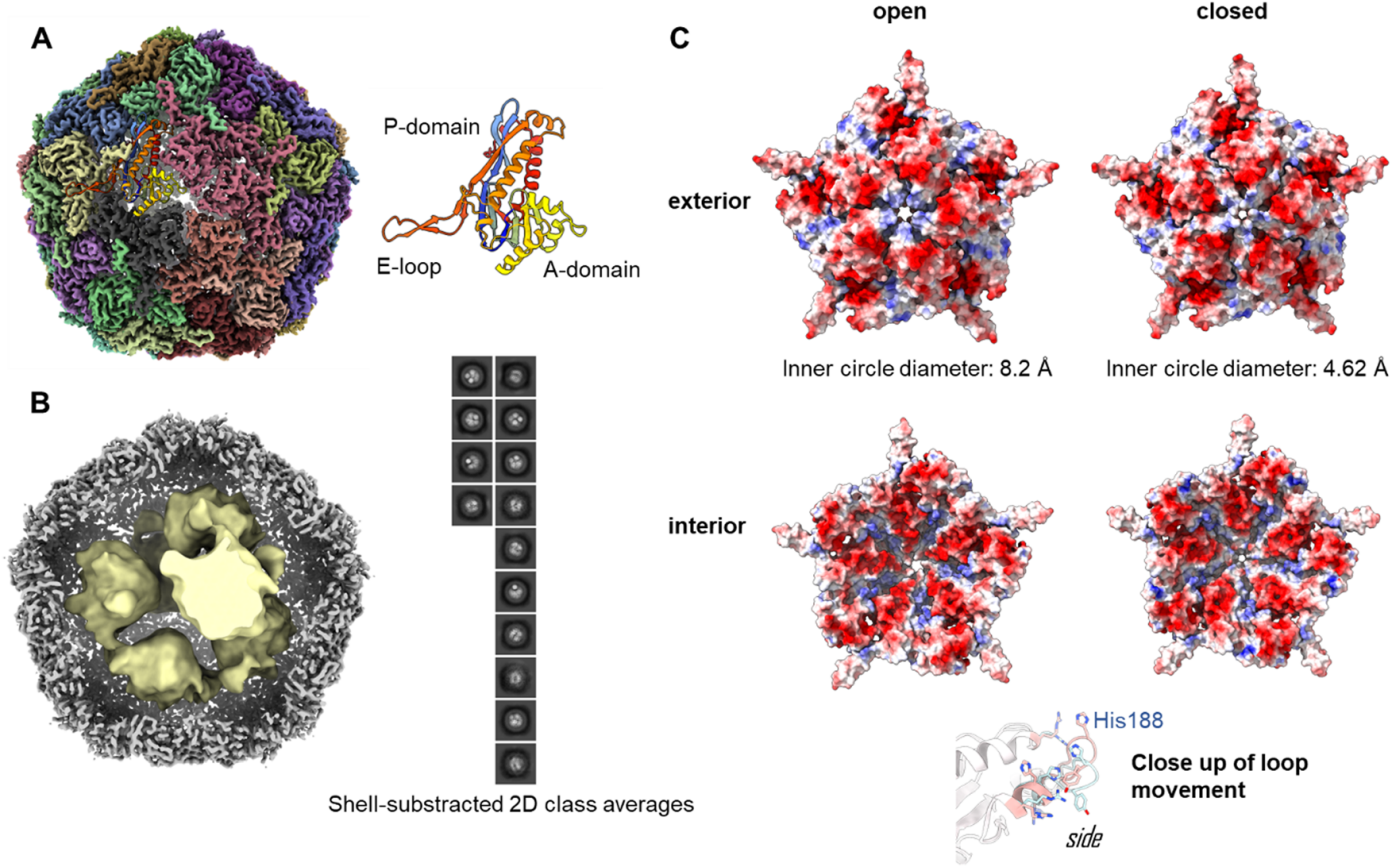
Cryo-EM analysis of Enc{tdNfsB}. (A) High resolution map with a single subunit shown as cartoon rendering. (B) A composite map of the high-resolution icosahedral encapsulin map (grey) and the interior map (yellow) resulting from particle subtraction of the cage densities and modelled density within the interior. Inset: 2-D classes following particle subtraction showing clear areas of density that correspond to the packing of tdNsfB within the encapsulin. (C) Five-fold pore in the open and closed states as determined by local refinement. The principle shift in pore size is reliant on the movement of His188 from an occluded position in the open to an extended-outwards position in the closed state.

To investigate the assembly of the Enc{tdNfsB} construct in detail, we performed single-particle cryo-EM analysis of the purified capsids (Fig. 2A). The overall resolution of the encapsulin icosahedral shell was determined to be 2.22 Å based on the gold standard Fourier Shell Correlation (FSC) 0.143 threshold (Fig. S2, ESI†).

The icosahedral reconstruction for the encapsulin shell closely matches those of previously published structures.^50,51^ A protein model was built into the map *de novo* and a single unit refined prior to the addition of symmetrical monomers to produce a final model. The calculated diameter of the shell was 24.0 nm, which correlates well with the expected solution hydrodynamic diameter and has a calculated internal volume of 3000 nm^3^.

To determine the loading of tdNfsB within the shell, particle subtraction was carried out on the final particle stack. The encapsulin cage was removed and particles were aligned in 2-D prior to a low resolution 3-D reconstruction. Discrete densities were visible in the 2-D classification and the 3-D reconstruction yielded six individual points of density when refined from *ab initio* modelling (Fig. 2B). Although we were unable to resolve the secondary structure of the subunits, we have established the presence of the cargo protein and can begin efforts to determine the precise stoichiometry. We observed density matching six tdNfsB dimers within the capsid though these could simply represent areas of high occupancy within the capsid and do not necessarily correlate directly. However, the same number of guest proteins per capsid was found in earlier studies from our group using inductively coupled plasma mass spectrometry (ICP-MS), super-resolution microscopy and fluorescent quantification of *M. smegmatis* engineered encapsulins, Enc{HaloTag-Ru^2+^}, Enc{HaloTag-AlexaFluor 647}, and Enc{eGFP}.^34,35^ Nevertheless, we employed gel-densitometry analysis to quantify the number of guest proteins within the capsid. Gel-densitometry analysis revealed ten to thirteen tdNfsB units per shell (Fig. S3, ESI†), which is ∼1.9 times more than the number identified through our cryo-EM analysis. We conclude that our analysis likely identified densities that correspond to tdNfsB units interacting with the inner surface of the encapsulin *via* electrostatic interactions.^48^ However, we cannot exclude the possibility that some tdNfsB units, which do not interact with the shell, remain unresolved in our cryo-EM analysis. This may be due to high flexibility and conformational heterogeneity of these units, preventing their clear resolution within the capsid's inner volume.

### Pores of the Enc{tdNfsB} complex

The crystal and cryo-EM structures of encapsulins from close homologs, have multiple openings in their capsid shells, including a threefold pore and an five-fold pore.^27,50–52^ These openings are thought to be the channels for the substrates and/or products of the cargo protein.

In our refined icosahedral model, the uncharged five-fold pore closely resembles that of the *M. smegmatis* dye-decolorizing peroxidase (DyP)-loaded encapsulin (PDB 7BOJ), with an inner circle diameter of 8.2 Å as measured by MoleOnline.^50^ The cryo-EM map in this area was ambiguous however and appears to be the superimposition of flexible states. To resolve this, the pentameric subunit was examined by 3-D classification and resulting volumes were refined using local refinement without symmetry constraints. This isolation a series of volumes that could be categorized as open, closed or uninterpretable. The open and closed particles were pooled and refined to yield complete, unambiguous maps (Fig. S2, ESI†). The closed pore inner circle diameter in this structure was 4.62 Å (Fig. 2C). The primary drive of this is a shift in the pore forming loop (measuring 3.84 Å between the displaced His188 alpha carbon, see attached movies M1 and M2). The underlying cause of this movement is unclear and may represent an equilibrium of states when resting at cellular pH or could be a result of different packing states within the particles stacks. A similar dynamic has been observed in the 5-fold pore of the iron storage encapsulin, Enc{Ftn}, from the halophilic bacterium *Haliangium ochraceum.*^53^

### Enzymatic activity of the encapsulated nitroreductase NfsB and tdNfsB

**Fig. 3:**
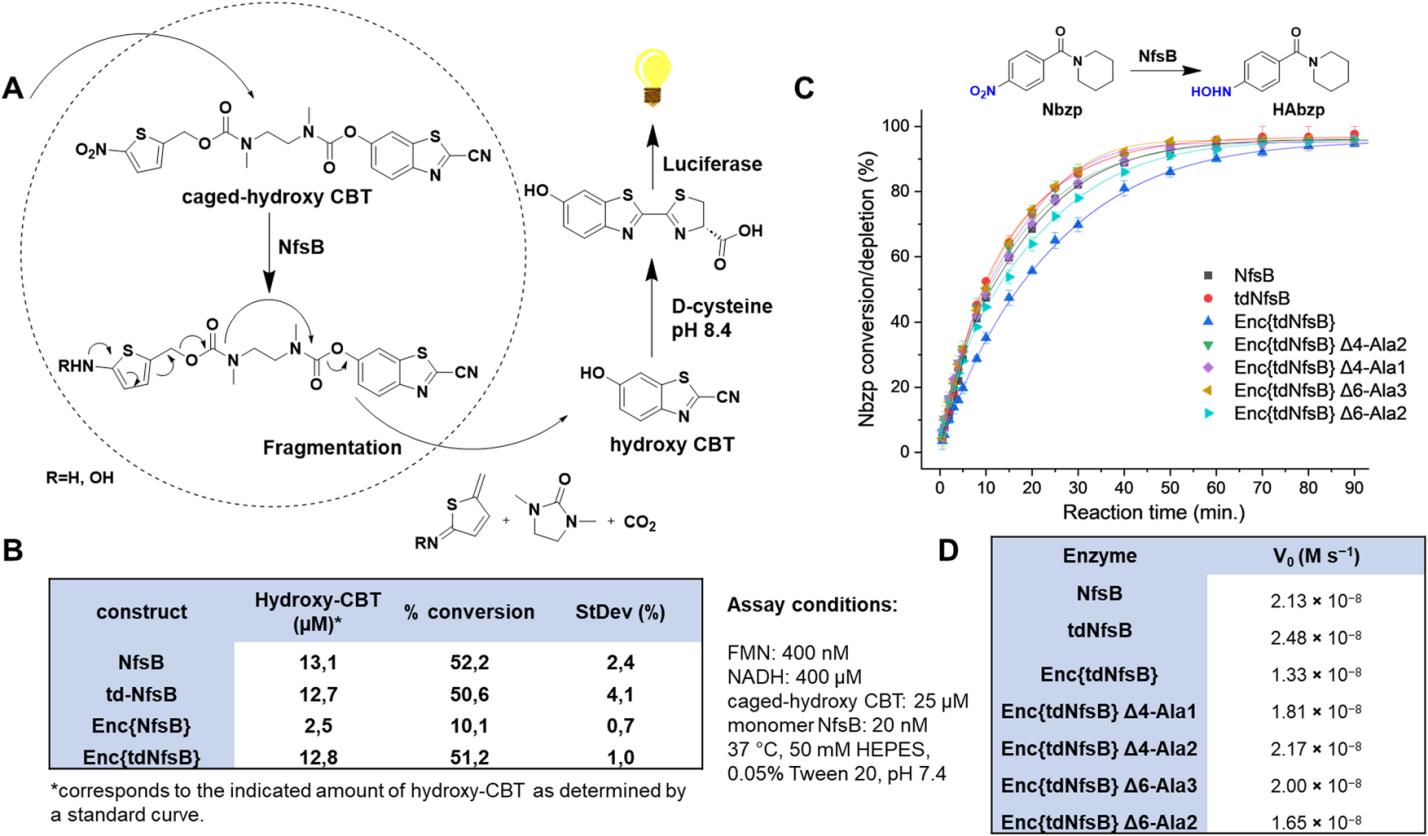
Enzymatic activity of Enc{NfsB} and Enc{tdNfsB}. (A) Schematic outline of the coupled luciferase assay using caged-hydroxy-CBT as a NfsB substrate. (B) Comparison of the enzymatic activity of NfsB, tdNfsB, Enc{NfsB} and Enc{tdNfsB} as analyzed by the coupled luciferase assay. The conversions shown have been normalized to a no-enzyme control. (C) HPLC analysis of Enc{NfsB}, Enc{tdNfsB}, four pore mutants of Enc{tdNfsB}, as well as NfsB and tdNfsB, in turning over the substrate Nbzp. Gray: NfsB; Red: tdNfsB; Blue: Enc{tdNfsB}; Green: Enc{tdNfsB} Δ4Ala2; Purple: Enc{tdNfsB} Δ4Ala1; Yellow: Enc{tdNfsB} Δ6Ala3; Turquoise: Enc{tdNfsB} Δ6Ala2. Assay conditions: FMN: 400 nM, NADH: 400 μM, Nbzp: 25 μM, enzyme: 20 nM tdNfsB or 3.33 nM Enc{tdNfsB} in 50 mM HEPES, 0.05% Tween 20, pH 7.4. The data points shown have been normalized to a no-enzyme control. (D) Reaction velocity *V*_0_ of the different constructs shown in (C).

To assess the enzymatic activity of Enc{tdNfsB} and Enc{NfsB} in comparison to their free forms, tdNfsB and NfsB, we devised a straightforward assay utilizing luciferase as a read-out combined with a NfsB bioluminescent reporter substrate, caged-hydroxy-CBT (6-hydroxy-2-cyanobenzothiazole, Fig. 3A).^4^ Caged-hydroxy-CBT is obtained by coupling a nitro-containing thiophenyl moiety (NfsB substrate) *via* a diamine linker to a luciferin substrate. Upon reduction of the nitro group to a hydroxylamine or amine, the molecule self-immolates and fragments to release hydroxy-CBT. Under basic conditions (pH 8.4), the liberated luciferin precursor hydroxy-CBT reacts with d-cysteine in a condensation reaction to form d-luciferin, which then undergoes enzymatic oxidation to an excited state, producing a measurable luminescent signal. This multi-step process is essential for generating the assay read-out. When analyzing encapsulated NfsB, the enzymatic and chemical reactions inside and outside the shell may be hindered by limited substrate or cofactor access due to the need for diffusion through the protein shell. Also, caged-hydroxy-CBT might accumulate inside the shell, resulting in higher initial substrate concentrations for the first reaction step. Similarly, limited diffusion of the product or fragmentation products out of the shell could lead to enzyme inhibition. Given that we supply substrate and cofactors at concentrations at least 20 times higher than the enzyme concentration, we believe substrate and cofactor access is not a limiting factor. Furthermore, the assay is designed as an end-point assay, and we have chosen a time point where only 50% of the substrate is converted. Hence, we believe that the assay is well suited to perform an initial screening on enzymatic activities of free and encapsulated enzyme.

This assay setup allowed us to track the pathway of the NfsB substrate into the capsid, its conversion by NfsB, subsequent diffusion out of the capsid, and its use as a luciferase substrate, leading to the emission of a bioluminescent signal. Furthermore, this assay format provided valuable insights into the catalytic activity of NfsB, an enzyme that relies on two cofactors, flavin mononucleotide (FMN) and reduced nicotinamide adenine dinucleotide (NADH).^43^ To quantify the assay, we generated a standard curve utilizing hydroxy-CBT (Fig. S4A, ESI†). Our experiments revealed that non-encapsulated NsfB and tdNfsB exhibited similar activity levels (see Fig. 3B). Specifically, we observed 50% conversion of the substrate caged-hydroxy-CBT after 60 min of incubation under the specified assay conditions. However, upon encapsulation, there was a notable decline in activity, particularly evident in the case of Enc{NfsB}, where only 10% conversion were observed. Conversely, Enc{tdNfsB} demonstrated substrate conversion comparable to those of the free enzymes NsfB and tdNfsB. By directly comparing Enc{NfsB} and Enc{tdNfsB}, where differing enzymatic activities due to shell effects can be largely excluded, we validate that monomeric NfsB is significantly less active than dimeric NfsB when encapsulated. Free NfsB and tdNfsB however showed similar conversion rates, showing that both enzymes are equally active in solution. This supports our initial hypothesis that dimer formation of monomeric NfsB within the confines of the capsid may be challenging.

Additionally, we conducted the assay with varying amounts of cofactors and screening concentrations ranging from 0 to 400 μM, and 0 to 400 nM for NADH and FMN, respectively. Lower concentrations of NADH affected the activity of Enc{tdNfsB}, while the free tdNfsB and NfsB remained unaffected within the tested concentration range (Fig. S4B, ESI†). This suggests that higher cofactor concentrations might be necessary to overcome potential bottlenecks in mass transport across the protein shell. Similar trends were observed for FMN, albeit less pronounced. Endogenous FMN is potentially already bound to NfsB (at least partially), and adding FMN externally is most likely saturating the binding site at the dimer interface and no further FMN co-factor diffusion is necessary throughout the assay. The gradual increase for Enc{tdNfsB} is again suspected to be a result of limiting diffusion through the protein shell. Again, from this assay, we inferred that NfsB may encounter difficulties in forming active dimers within the protein shell. Consistent with this notion, the results of the assay with varying FMN concentrations indicate that only Enc{tdNfsB} can effectively bind FMN, a process occurring at the dimer interface.^54^ Conversely, increasing FMN concentration had no discernible effect on the activity of Enc{NfsB}. This underscores the advantage of using tdNfsB as cargo, as it ensures the encapsulation of functionally active enzyme dimers within the encapsulin structure. It is plausible that once incorporated within the encapsulin, monomeric NfsB is positioned too far apart to facilitate functional dimer formation, and dimerization may be low outside the encapsulin due to the high expression levels of both NfsB and encapsulin. Expression under alternative promoters or reduced induction levels could potentially promote dimer formation outside encapsulin from the monomeric construct, prior to incorporation into encapsulin as a dimer. Finally, the optimal molar ratio of NfsB/tdNfsB and FMN as 1 : 40, as well as NfsB/tdNfsB and NADH as 1 : 40 000, was established for subsequent assays. Enc{NfsB} was excluded from further analysis due to its apparent lack of enzymatic activity.

To validate our findings through a direct assay, we employed a high-performance liquid chromatography (HPLC) format utilizing a conventional NfsB substrate, (1-(4-nitrobenzoyl)piperidine, Nbzp).^5^ Nbzp has been identified as a promising prodrug candidate potentially activated by NfsB. The reaction between Nbzp and NfsB was monitored, capturing a time-dependent profile of substrate depletion. Analysis of the HPLC results revealed a conversion rate of ≥ 60% of the parent compound within 50 min, with the primary reaction product identified as 1-(4-hydroxylaminobenzoyl)piperidine (HAbzp). Both free enzymes, NfsB and tdNfsB, exhibited comparable substrate conversion rates throughout the recorded timeframe (Fig. 3C). Interestingly, in the direct HPLC assay, encapsulated tdNfsB exhibited a slightly decreased initial reaction velocity *V*_0_ (Fig. 3D), however was able to convert the same concentration of Nbzp within the observed timeframe, as compared to free enzyme. Potentially, this effect stems from hindered substrate diffusion towards the interior of encapsulin.

We subsequently determined enzyme kinetics using varying concentrations of the substrate Nbzp (Fig. S4, ESI†). Cofactor turnover was monitored by measuring NADH absorbance at 340 nm. Fitting the data to the Michaelis–Menten model yielded *k*_cat_ values of 11.2 s^−1^ for free tdNfsB and 6.75 s^−1^ for encapsulated tdNfsB. The *K*_M_ values were determined to be 3.71 × 10^−4^ M for free tdNfsB and 3.21 × 10^−4^ M for Enc{tdNfsB}. These results demonstrate that encapsulating tdNfsB supports high catalytic activity, but that encapsulated enzyme is slightly less active in catalysis. This might be due to close packing or interactions with the interior of the encapsulin shell leading to less flexibility of the enzyme. Similarly, product diffusion out of the encapsulation matrix may be slower, potentially leading to product inhibition effects that reduce *k*_cat_. Interestingly, encapsulated tdNfsB and free enzyme exhibited similar *K*_M_ values, this suggests that encapsulation primarily affects the catalytic step rather than the binding step. However, this effect may vary with different substrates. Nbzp, a small molecule with a molecular weight of 234.3 Da, might have minor diffusion limitations. In contrast, other studies, such as those by T. Giessen's group or also by our group, have reported increased Michaelis constants for encapsulated enzymes or metal catalysts, particularly in systems loaded with larger substrates.^34,55^

In order to show the general applicability of Enc{tdNfsB} in reducing nitroaromatic compounds, we employed a number of known nitroreductase substrates in the above assay (MA60, AV2, AV4, MA63,^4,42^ CB1954,^41^ and CoNO_2_,^56^ Fig. S4C and Table S2, ESI†). The primary goal of this experiment is to demonstrate that encapsulated tandem nitroreductase remains active in reducing a variety of structurally diverse molecules. Firstly, we tested the product conversion of all selected candidates after 30 minutes of incubation with free tdNfsB. The identity of the reaction products was confirmed through mass spectrometry analysis. All molecules were converted by the tandem enzyme, validating that not only the free NfsB dimers, but also the fused dimeric NfsB is active. As expected, the different molecules exhibited varying conversion rates, likely due to their different affinities towards NfsB.^57^ Apart from varying affinity to the enzyme, different physicochemical properties such as the reduction potential of the nitro group potentially also influence conversion rates. Upon investigating substrate conversion with Enc{tdNfsB}, we observed conversion of all molecules, with slight differences between the encapsulated and free tdNfsB. Notably, due to the distinct molecular structures, the ability of each molecule to diffuse into the capsid potentially varies. Consequently, depending on the substrate, conversion rates may be higher with either the free or encapsulated enzyme. Higher conversion rates within the capsid are most likely due to substrate accumulation, which enhances enzymatic turnover in the confined space. Interestingly, the two molecules AV2 and AV4 (see Table S2, ESI†), which both carry a potentially charged piperazine moiety under the applied conditions, exhibited significantly higher conversion rates within Enc{tdNfsB}. This increase in conversion may be due to electrostatic interactions within the 5-fold pore region, potentially enhancing diffusion rates and accumulation of AV2 and AV4 inside the capsid.

### Construction of pore mutants of Enc{tdNfsB}

**Fig. 4:**
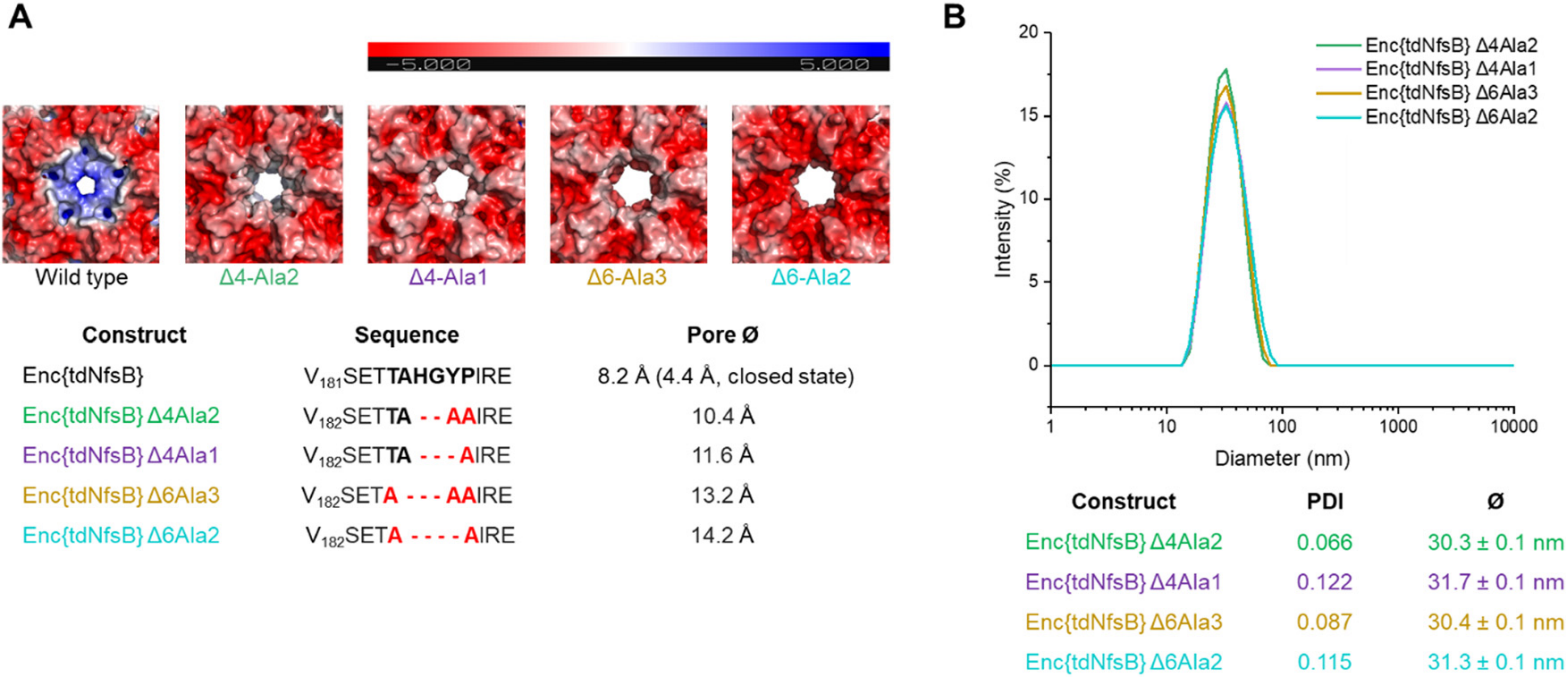
Design and characterization of four pore mutants of Enc{tdNfsB}. (A) Exterior view of the 5-fold pores of encapsulin shell of *M. smegmatis* shown in electrostatic surface representation. Surface representations were generated using APBS electrostatics plugin in PyMOL. Red color represents negative charge potential (−5 eV), while blue color represents positive charge potential (5 eV). We show computational models of surface representations. (B) DLS analysis of the pore mutants.

Since we have observed a potential hindered substrate access to Enc{tdNfsB} in our above kinetic analysis, we proposed that pore diffusion of the substrate or the NfsB co-factors are a limiting factor and slowed down the reaction, especially at low substrate concentrations. Hence, we constructed four encapsulin mutants and increased the pore size of the five-fold pore by removing six amino acids in the pore-forming loop by site-directed mutagenesis (see ESI†). Encapsulin is built from 60 identical monomers, and five monomers that form a pentameric structure give rise to one five-fold pore, twelve of which exist per capsid. As was shown previously, the 5-fold loop forming region can tolerate amino acid substitutions and deletions without affecting self-assembly and stability of the encapsulin shell.^44,47,58,59^ Furthermore, pore-engineering has been recently shown to enhance small molecule flux in a *T* = 3 encapsulin system from *Myxococcus xanthus* (*M. xanthus*).^60^ Encapsulin variants shown in Fig. 4A were generated. Constructs were cloned and purified as reported for the wild-type encapsulin. The four mutant constructs were systematically analyzed and characterized using Superose™6 chromatography, BN-PAGE, SDS-PAGE, DLS, and TEM (Fig. 4 and Fig. S5, ESI†). The results indicated that all mutant constructs exhibited behavior comparable to that of the wild-type capsid, displaying similar retention times, polydispersity indices, hydrodynamic radii, diameters, and structural assembly characteristics. Computational models of encapsulin variants Δ4-Ala2, Δ4-Ala1, Δ6-Ala3, Δ6-Ala2 estimated enlarged pore diameters of 10.4 Å, 11.6 Å, 13.2 Å, and 14.2 Å, respectively. Subsequently, we employed these mutants and determined their effects on the enzymatic activity of encapsulated tdNfsB. A total increase in conversion of Nbzp after 30 min by 12.8% (*p* ≤ 0.01), 16.7% (*p* ≤ 0.05), 16.7% (*p* ≤ 0.01), and 8.2% (n.s.) was observed for EncΔ4-Ala2{tdNfsB}, EncΔ4-Ala1{tdNfsB}, EncΔ6-Ala3{tdNfsB}, and EncΔ6-Ala2{tdNfsB}, respectively (Fig. 3C), in comparison to Enc{tdNfsB}. Kinetic analysis of the two pore mutants EncΔ4-Ala1{tdNfsB} and EncΔ6-Ala3{tdNfsB} gave *K*_M_ values of 5.66 × 10^−4^ M and 3.67 × 10^−4^ M, as well as *k*_cat_ values of 16.61 s^−1^ and 17.93 s^−1^, respectively (Fig. S4, ESI†). This suggests that the decreased catalytic activity of the encapsulated enzyme can be improved upon pore modulation. Given the absence of a clear correlation between pore size and conversion rate, the enhancement may also be influenced by the electrostatic properties of the pore surface. As illustrated in Fig. 4A, all pore mutants exhibit a neutral to negative (red) charge in the pore region, in contrast to the positively charged wild-type pore. Therefore, in addition to pore size, which may only affect substrate diffusion up to a certain threshold, electrostatic interactions between the substrate and pore surface could also be a limiting factor. However, the observed increase in turnover across all four mutants may also arise from other mechanisms, such as the introduction of assembly defects *via* the mutations that enhance substrate access or differences in enzyme cargo loading within the mutant cages.

### Activity of Enc{tdNfsB} vs tdNfsB in cellular environments

**Fig. 5:**
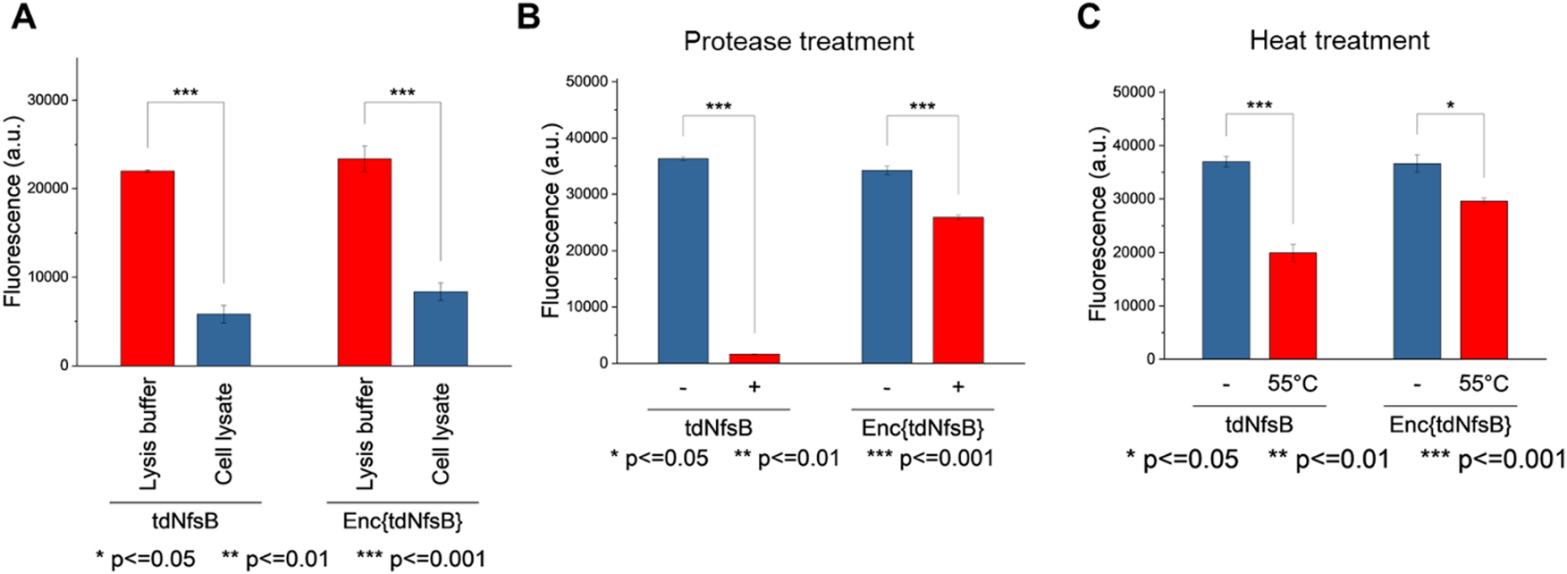
Comparison of Enc{tdNfsB} and free tdNfsB in terms of stability and enzymatic activity under challenging conditions. The profluorphore CoNO_2_ was applied as a nitroreductase substrate. (A) Enzymatic activity in lysis buffer and cell lysate. Incubation time with CoNO_2_ was 10 min. (B) Enzymatic activity in the presence and absence of protease. Treatment time with protease was 10 min, followed by a 10 min incubation with CoNO_2_. (C) Enzymatic activity after heat treatment at 55 °C for 1 min, followed by a 10 min incubation with CoNO_2_.

The application of nitroreductase enzymes encompasses a wide array of scenarios, often requiring robust enzymatic activity under diverse and challenging conditions. These conditions include elevated temperatures, cellular lysates, extracellular matrices, blood samples, immobilization on solid supports, and exposure to organic solvents. In our study, we aimed to investigate whether encapsulating NfsB within a protein cage could enhance its performance. Initially, we assessed the efficacy of Enc{tdNfsB} compared to free tdNfsB under various conditions, including exposure to protease, heat, cell lysate, and in lysis buffer. We used fluorescence read-out with the profluorophore 7-(diethylamino)-3-nitro-2*H*-chromen-2-one (CoNO_2_), which NfsB converts to the fluorophore 3-amino-7-(diethylamino)-2*H*-chromen-2-one (CoNH_2_), as a reliable method to quantify NfsB activity. Similar activity levels were observed between Enc{tdNfsB} and tdNfsB in Pierce™ IP lysis buffer or cell lysates (Fig. 5A). Fluorescence detection of CoNO_2_ in the cell lysate exhibited approximately one-third of the signal intensity observed in the buffer-only condition. Increasing the concentration of NADH by ∼4-fold did not significantly enhance the signal, suggesting that the reduced fluorescence may result from quenching by cellular components released during lysis, such as nucleic acids. This quenching effect is consistent with the behavior commonly observed for coumarin dyes.^61^ However, Enc{tdNfsB} exhibited a remarkable increase in enzymatic activity compared to tdNfsB in the presence of protease (Fig. 5A and B). While the free enzyme was completely inactive after a 10-minute incubation, the encapsulated enzyme maintained comparable activity to the non-treated condition. Distinct protease degradation of free vs encapsulated enzyme could be observed by SDS-PAGE (Fig. S6, ESI†). This highlights the significant beneficial effect of encapsulation on tdNfsB activity, particularly under challenging conditions, and our results align with previous findings on a *T* = 3 capsid from *M. xanthus* or *T* = 1 encapsulins from *Thermatoga maritima* or *Rhodococcus erythropolis*.^28,31,60^ A similar but less pronounced effect was observed upon heat treatment of both free and encapsulated NfsB. Encapsulation slightly improved enzymatic activity.

**Fig. 6:**
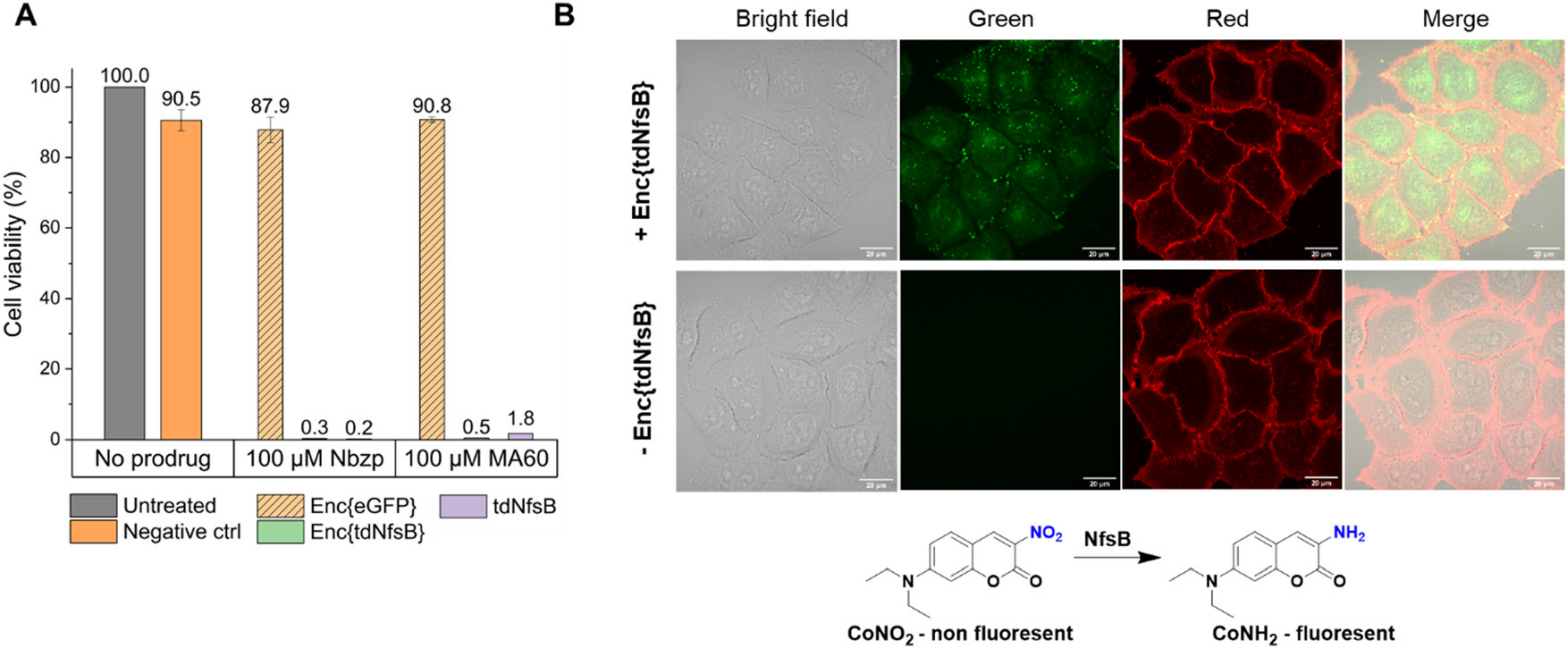
Enc{tdNfsB} activation of a nitroaromatic prodrug and a profluorophore in live cells. (A) Effect of extracellular prodrug activation on H1299 lung carcinoma cell viability. (B) Fluorescence microscopy and bright-field images of HeLa cells treated for 2 h with either 40 μM CoNO_2_, 400 μM NADH, 400 nM FMN, or 40 μM CoNO_2_, 400 μM NADH, 400 nM FMN, and 20 nM Enc{tdNfsB}. Fluorescence microscopy images of HeLa cells treated with Enc{tdNfsB} show green fluorescence from uncaged CoNH_2_.

Subsequently, we investigated whether Enc{tdNfsB} retained its activity on living cells, specifically its ability to convert the prodrug Nbzp into its active form for subsequent cell-killing activity. Co-administration of Enc{tdNfsB} and the prodrug Nbzp to pre-seeded lung carcinoma cells (H1299) resulted in complete loss of cell viability compared to controls utilizing encapsulated eGFP (enhanced green fluorescent protein), Enc{eGFP},^34^ which confirms the efficient conversion of Nbzp into its active metabolite and subsequent uptake by H1299 cells. A similar decrease in cell viability was observed with the non-encapsulated tdNfsB control, indicating that encapsulated nitroreductase functions effectively as a surrogate for free tdNfsB. The encapsulation offers additional benefits, such as enhanced stability (Fig. 5) and the potential for shell modifications, enabling *e.g.* cell type-specific targeting.^37^ This hypothesis was supported further by cell-assays using the profluorophore CoNO_2_. Upon co-administration of Enc{tdNfsB} and CoNO_2_ to pre-seeded HeLa cells (human cervical cancer cells), fluorescence accumulation within the cells was evident after two hours of incubation, which confirms the conversion of CoNO_2_ and subsequent uptake of CoNH_2_ by HeLa cells (Fig. 6). Furthermore, our experiments indicated that Enc{tdNfsB} exhibited no toxicity towards HeLa or HT1299 cells.

## Conclusion

We have successfully demonstrated the encapsulation of an active NfsB enzyme that relies on two co-factors for its activity. This achievement involved cloning a tandem enzyme to facilitate dimer formation, which is essential for co-factor binding, and subsequent activity. Through thorough characterization using TEM, DLS, and cryo-EM, we gained deep insights into the protein assembly. Moreover, our investigation revealed that the increasing pore size of the encapsulation system could enhance enzyme turnover, possibly due to less restricted diffusion whilst maintaining the protective encapsulated environment. The encapsulated nitroreductase exhibited activity on various structurally distinct substrates and demonstrated improved performance compared to the free enzyme, particularly under challenging conditions. Furthermore, encapsulated NfsB efficiently activated prodrugs and profluorophores in the extracellular environment. In the next phase of our research, we plan to introduce a targeting functionality onto the protein cage, enabling specific tissue targeting. Additionally, we aim to explore the possibility of co-encapsulating a NAD-regenerating enzyme such as glucose dehydrogenase (GDH).^62^ Previous studies have shown the feasibility of co-encapsulating different enzymes within protein nanocages. Overall, our findings contribute to a deeper understanding of enzyme encapsulation and hold promise for stabilizing enzymes for various applications including diagnosis, prodrug activation, and beyond.

## Author contributions

Conceptualization: M. Z. and C. JT.; methodology: M. Z., B. D. K. S., HG. K., L. K., M. G., K. S. and M. J. C.; software: M. J. C.; validation: M. Z., M. J. C., C. JT., R. S. M. J., W. S., A. M., and J. K.; resources: C. JT., R. S., M. J., W. S., A. M. HG. K. and J. K.; data curation: M. J. C. and M. Z.; writing – original draft preparation: C. JT.; writing – review and editing: M. J. C., M. Z., J. K., R. S., M. J., W. S., A. M., B. D. and C. JT. All authors have read and agreed to the published version of the manuscript.

## Conflicts of interest

There are no conflicts to declare.

## Supplementary Material

CB-006-D4CB00127C-s001

CB-006-D4CB00127C-s002

CB-006-D4CB00127C-s003

## Data Availability

The data supporting this article have been included as part of the ESI.†
