# Supplementary material for: A nanoengineered tandem nitroreductase: designing a robust prodrug-activating nanoreactor
Source: RSC Chem Biol. 2024 Nov 4;6(1):21–35. doi: 10.1039/d4cb00127c (PMC11532998; doi:10.1039/d4cb00127c)
Supplement: CB-006-D4CB00127C-s003 [file CB-006-D4CB00127C-s003.pdf]

Supplementary Information (SI) for RSC Chemical Biology.
This journal is © The Royal Society of Chemistry 2024

Supporting Information

A Nanoengineered Tandem Nitroreductase: Designing a Robust Prodrug-Activating Nanoreactor

Contents

Supporting figures ... 2
Chemicals, analytics and general remarks ... 10
Genetic engineering ... 10
Cloning of pETDuet-1: Strep-Tag-NfsB (MCS2) ... 10
Cloning of pETDuet-1: Enc-Strep-Tag (MCS1); NfsB (MCS2) ... 10
Cloning of pETDuet-1: Enc-Strep-Tag (MCS1); eGFP (MCS2)[3] ... 11
Construction of five-fold pore encapsulin mutants ... 11
Table S1. Primers and oligonucleotides used in this study. ... 11
Complete protein sequences ... 11
Table S2. Molecular structures of compounds applied in this study. ... 12
Table S3. Refinement statistics. ... 13
References ... 14

Supporting figures

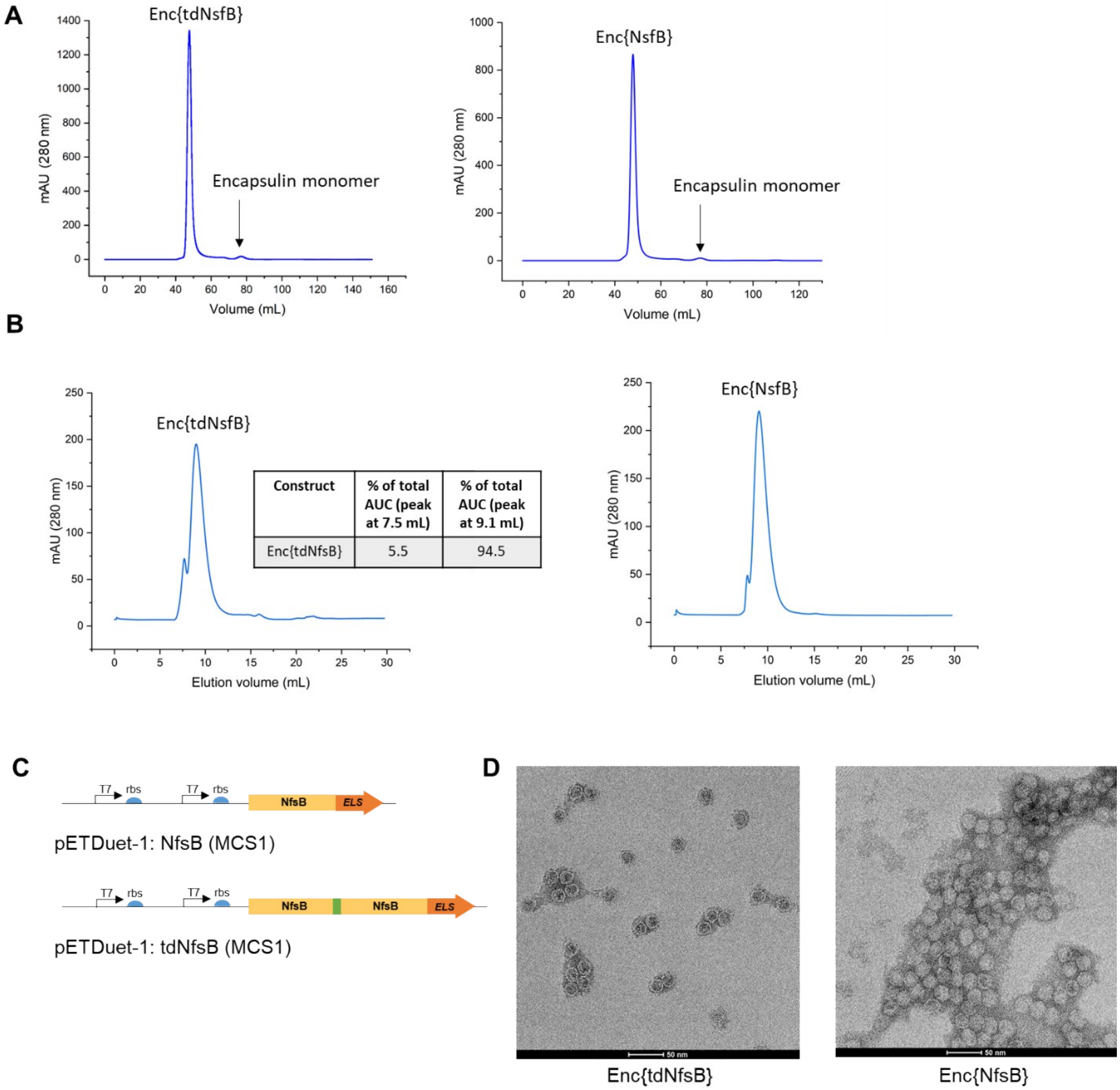

Figure S1: Design and characterization of NfsB, tdNfsB, Enc{NfsB} and Enc{tdNfsB}. A) Representative chromatograms of size-exclusion column chromatography runs (HiLoad 16/600 Superdex 200 PG column). UV: 280 nm. Each encapsulin capsid peak was collected and further analyzed by DLS. B) Representative chromatograms of size-exclusion column chromatography runs on a Superose™ 6 Increase 10/300 column. UV: 280 nm. C) Schematic outline of the cloned constructs of NfsB and tdNfsB. D) TEM analysis of Enc{NfsB} and Enc{tdNfsB}, as eluted from the HiLoad Superdex 200 PG column. Bar: 50 nm.

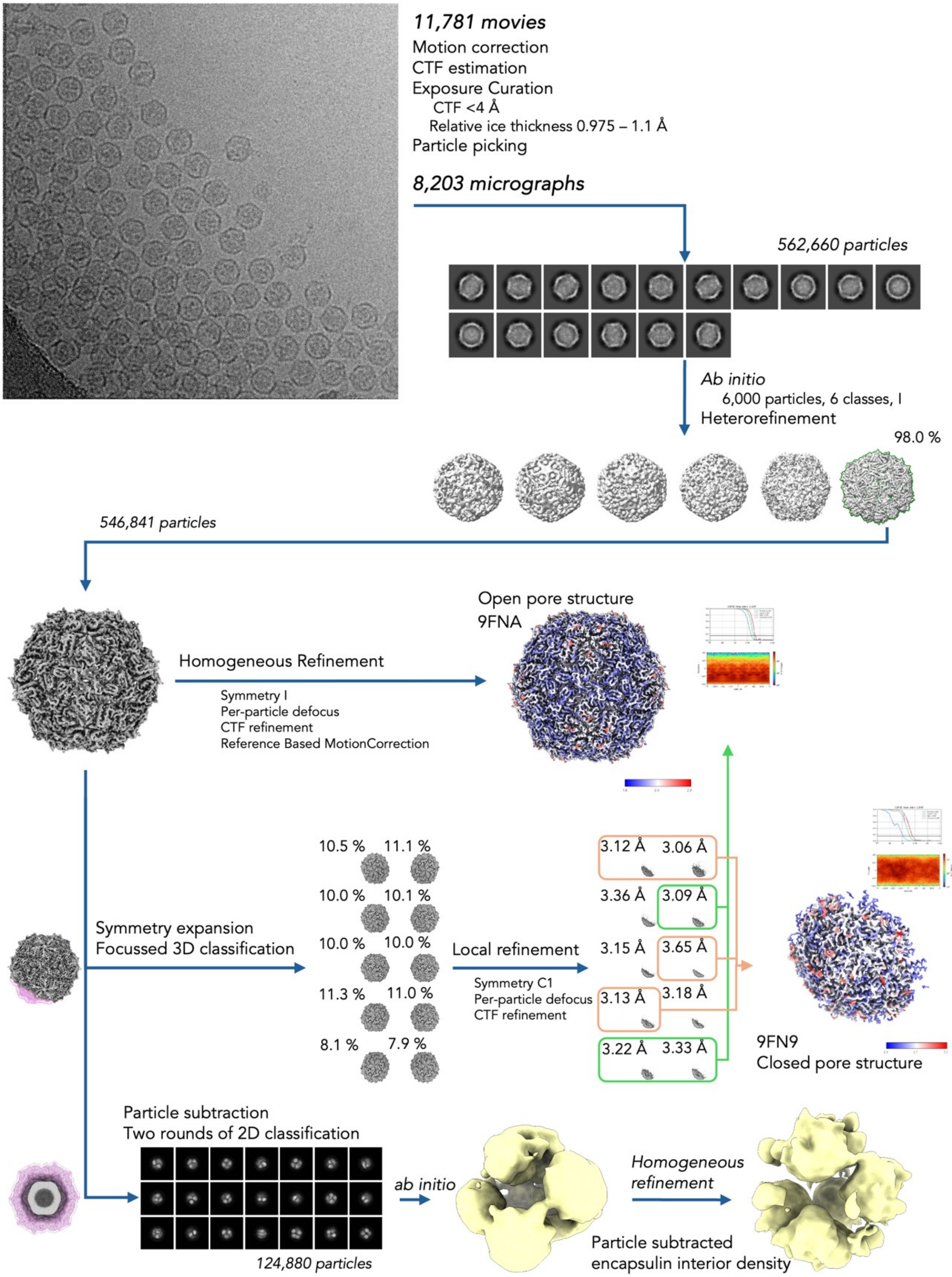

11,781 movies
Motion correction
CTF estimation
Exposure Curation
CTF <4 Å
Relative ice thickness 0.975 – 1.1 Å
Particle picking
8,203 micrographs
562,660 particles
Ab initio
6,000 particles, 6 classes, I
Heterorefinement
98.0 %
546,841 particles
Open pore structure
9FNA
Homogeneous Refinement
Symmetry I
Per-particle defocus
CTF refinement
Reference Based MotionCorrection
10.5 %
11.1 %
10.0 %
10.1 %
10.0 %
10.0 %
11.3 %
11.0 %
8.1 %
7.9 %
Symmetry expansion
Focussed 3D classification
Local refinement
Symmetry C1
Per-particle defocus
CTF refinement
3.12 Å
3.06 Å
3.36 Å
3.09 Å
3.15 Å
3.65 Å
3.13 Å
3.18 Å
3.22 Å
3.33 Å
9FN9
Closed pore structure
Particle subtraction
Two rounds of 2D classification
124,880 particles
ab initio
Homogeneous refinement
Particle subtracted encapsulin interior density

Figure S2: Complete cryo-EM data processing workflow including a representative micrograph and resulting volumes with local resolutions. All data were processed to produce the icosahedral volume with the pore in an open state. Subsequent focused classification and refinement around the five-fold symmetry axis confirmed this state and identified the closed pore which was independently refined and a model produced. Efforts to determine the stoichiometry of the internal components used particle subtraction before multiple rounds of 2D-classification prior to *ab intio* model production and low-resolution refinement.

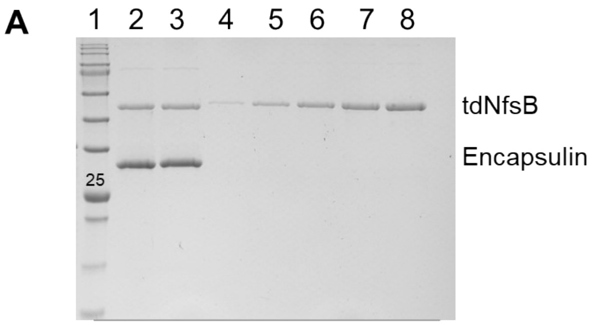

Lane	Sample
1	MW Ladder (kDa)
2	Enc{tdNfsB}, 2.5 µg
3	Enc{tdNfsB}, 2.5 µg
4	tdNfsB, 0.13 µg
5	tdNfsB, 0.39 µg
6	tdNfsB, 0.65 µg
7	tdNfsB, 0.92 µg
8	tdNfsB, 1.18 µg

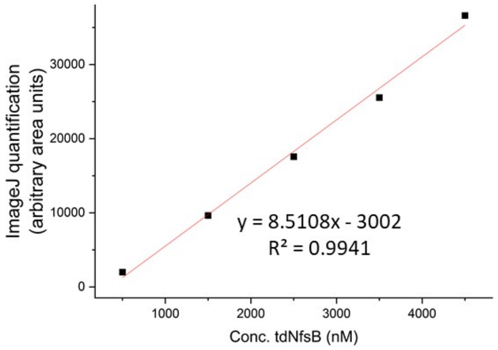

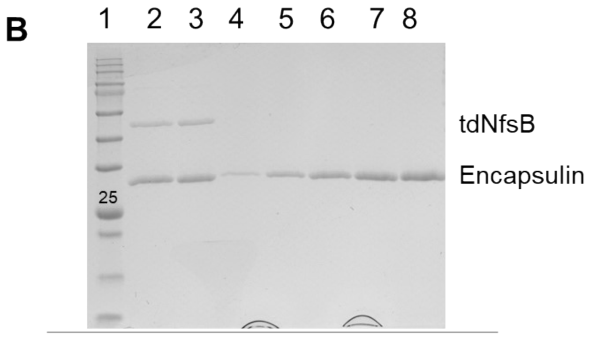

Lane	Sample
1	MW Ladder (kDa)
2	Enc{tdNfsB}, 2.5 µg
3	Enc{tdNfsB}, 2.5 µg
4	Enc (empty shell), 0.45 µg
5	Enc (empty shell), 1.13 µg
6	Enc (empty shell), 1.8 µg
7	Enc (empty shell), 2.48 µg
8	Enc (empty shell), 3.15 µg

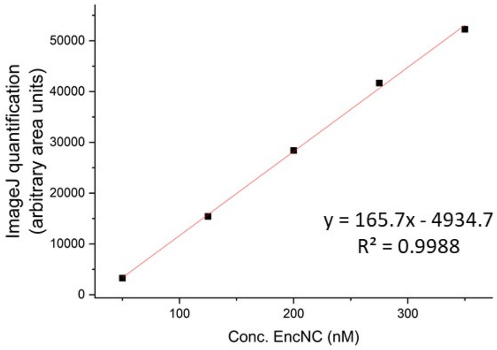

Figure S3: Gel-densitometry to quantify number of guest proteins. A) and B) show two representative SDS-PAGE gels of samples Enc{tdNfsB} and tdNfsB, as well as empty encapsulin shell analyzed for quantification purposes. Standard curves for subsequent protein quantification after Coomassie staining were plotted in Origin.

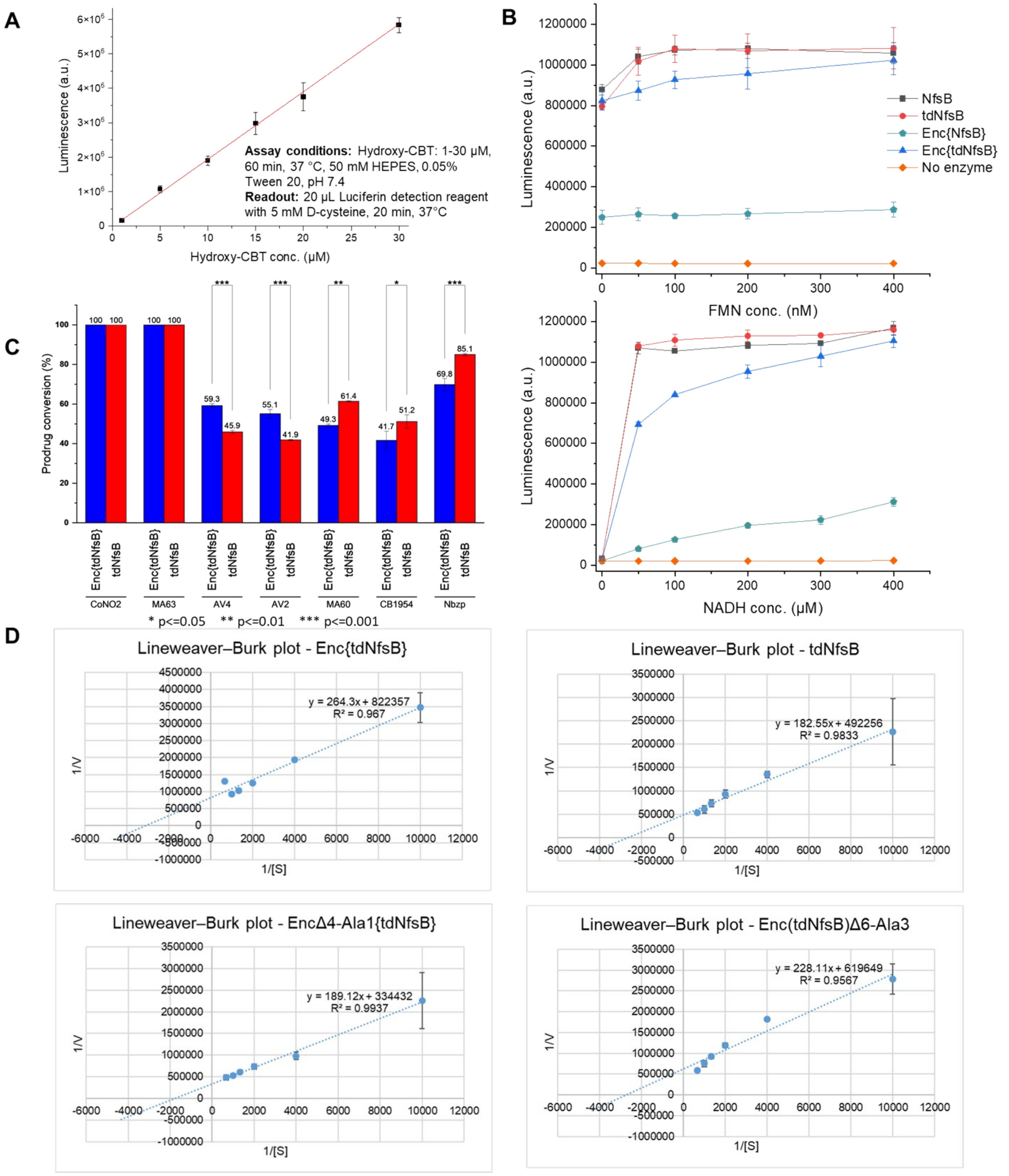

Figure S4: Enzymatic activity of Enc{NfsB} and Enc{tdNfsB}. A) Standard curve to quantify Hydroxy-CBT concentrations in the coupled luciferase assay. B) Screening of optimal NADH and FMN cofactor concentrations. Assay conditions: FMN: 0-400 nM. NADH: 0-400 µM, Hydroxy-CBT: 25 µM, enzyme: 10 nM tdNfsB or 3.33 nM Enc{tdNfsB}, 50 mM HEPES, 0.05% Tween 20, pH 7.4. Assay readout: Addition of 20 µL of Luciferin detection reagent (Promega, Germany), supplemented with D-cysteine to a final

concentration of 5 mM. After 20 min of incubation, bioluminescence was determined on a micro plate reader (Tecan). C) Several substrates converted by tdNfsB and Enc{tdNfsB}. D) Lineweaver–Burk plots to determine V_{max}, K_M and K_{cat} values of tdNfsB, Enc{tdNfsB} and the pore mutants Enc{tdNfsB} Δ6-Ala3 and Enc{tdNfsB} Δ4-Ala1. [S] is substrate (Molar), V is velocity (seconds).

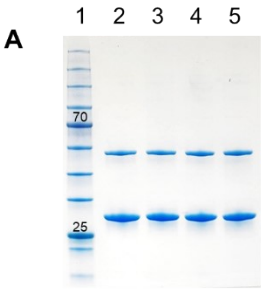

Lane	Sample
1	MW Ladder (kDa)
2	Enc{tdNfsB} Δ4Ala2
3	Enc{tdNfsB} Δ4Ala1
4	Enc{tdNfsB} Δ6Ala3
5	Enc{tdNfsB} Δ6Ala2

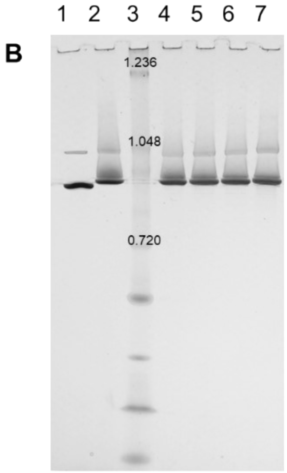

Lane	Sample
1	Enc{NfsB}
2	Enc{tdNfsB}
3	MW Ladder (MDa)
4	Enc{tdNfsB} Δ4Ala2
5	Enc{tdNfsB} Δ4Ala1
6	Enc{tdNfsB} Δ6Ala3
7	Enc{tdNfsB} Δ6Ala2

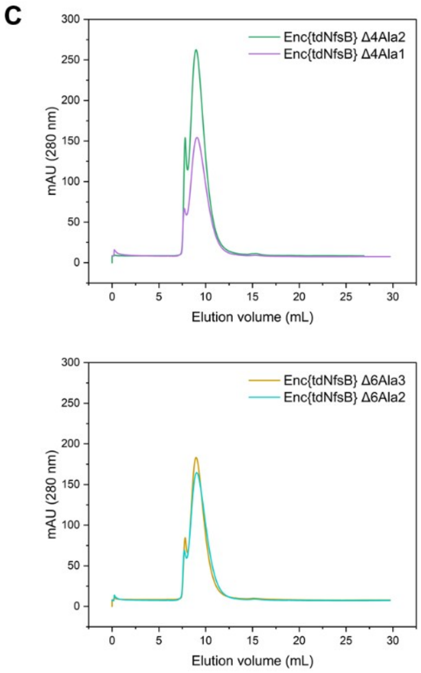

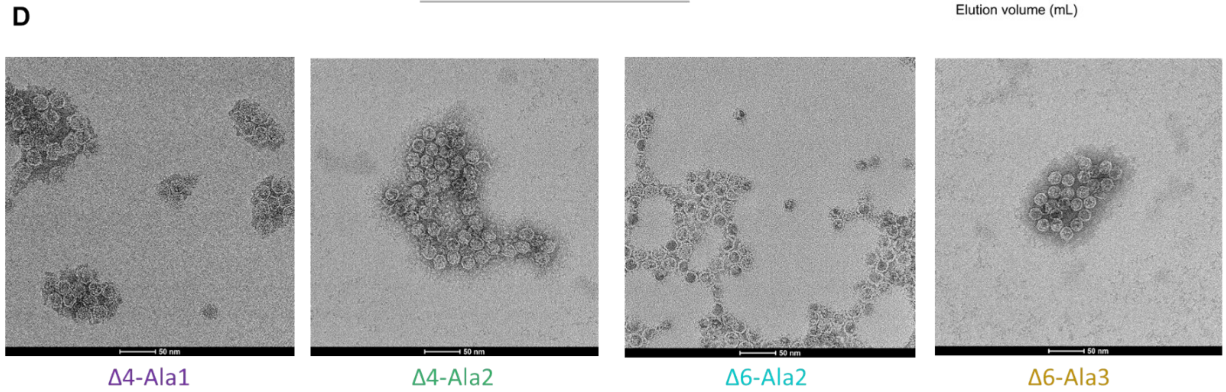

Figure S5: Characterization of four pore mutants of Enc{tdNfsB}. A) SDS-PAGE and B) BN-PAGE analysis after purification via affinity chromatography on a StrepTrap HP column, followed by size exclusion chromatography on a HiLoad 16/600 Superdex 200 PG column. C) Analysis of the four mutants by size-exclusion chromatography on a Superose™ 6 Increase 10/300 column. Semi-quantitative analysis using a generated standard curve with proteins ranging from 669000 Da to 13700 Da in size indicates a theoretical size of 1.23 MDa at 9.1 mL elution volume.

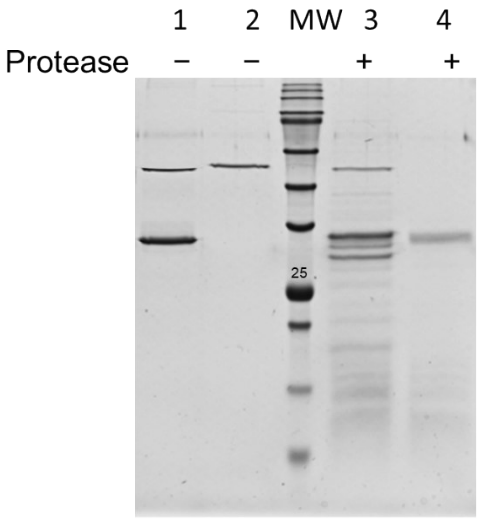

Lane	Sample
1	Enc{tdNfsB}
2	tdNfsB
MW	MW Ladder (kDa)
3	Enc{tdNfsB} + protease
4	tdNfsB + protease

Figure S6: SDS-PAGE analysis of samples treated with protease from *Streptomyces griseus*. The incubation was conducted using 1 U of protease per 200 pmol of tdNfsB at 37°C for 10 minutes in 50 mM HEPES, pH 7.4. Following incubation, aliquots were taken and further analyzed. SDS-PAGE analysis clearly shows complete degradation of free tdNfsB, while encapsulated tdNfsB is mostly preserved.

Chemicals, analytics and general remarks
Reagents were purchased from commercial suppliers (Sigma-Aldrich, Carl Roth, ChemCruz, Angene Chemicals, abcr, Promega) and used as received, unless noted otherwise. Ampicillin, lysozyme, dithiothreitol (DTT), phenylmethylsulfonyl fluoride (PMSF), phenazine-methosulfate (PMS), dimethyl sulfoxide (DMSO), trifluoroacetic acid (TFA), hydroxy-CBT, flavin mononucleotide (FMN), β-Nicotinamide adenine dinucleotide (NADH) and Protease from *Streptomyces griseus* were acquired from Sigma-Aldrich, Germany. Acetonitrile (ACN), HEPES, Tris-HCl, sodium chloride (NaCl), Tween 20 and ethylenediaminetetraacetic acid (EDTA) were purchased from Carl Roth, Germany. D-cysteine and the prodrug CB1954 were purchased from ChemCruz, while $CoNO_2$ was procured from Angene Chemicals. Prodrug Nbzp was obtained from abcr, Germany. CellTiter 96 AQueous MTS Reagent Powder and Luciferin Detection Reagent were purchased from Promega, Germany. HPLC chemicals and solvents were obtained in analytical grade and used as received.

Synthesis of caged hydroxy-CBT, MA60, MA63, AV2, and AV4 is published elsewhere.[1,2]

The pETDuet-1 plasmid used for cloning was obtained from Novagen, Merck. Restriction enzymes and the NEBuilder HiFi DNA Assembly kit were sourced from New England Biolabs, USA. CellMask Orange plasma membrane stain was obtained from ThermoFisher Scientific, Germany.

Strains *E. coli* XL1-Blue and *E. coli* BL21 Star (DE3) were obtained from Stratagene/Agilent and from Novagen, Merck, respectively.

Encapsulin: UniProt ID: I7G8Y9, Organism: *Mycobacterium smegmatis* mc^2155, Other descriptors: 29 kDa antigen CFP29, MSMEI_5672

ELS amino acids sequence (as published earlier in Lohner et al.[3]): Ser-Leu-Gly-Ile-Gly-Ser-Leu-Lys-Gly-Thr-Arg.

Genetic engineering

Cloning of pETDuet-1: Strep-Tag-NfsB (MCS2)
The commercially available pETDuet-1 vector (Novagen) was modified to carry a Strep-tag II sequence. Strep-tag II was introduced into MCS2 (MCS: multiple cloning site) using an annealed oligonucleotide cloning approach with primer pair MZ1_fw/ MZ2_rev (as indicated in Table S1), inserted between NdeI and FseI restriction sites. Similarly, the C-terminal ELS sequence was inserted between XhoI and PacI restriction sites using primer pair MZ3_fw/ MZ4_rev. Standard cloning techniques were then employed to insert the *E. coli* nitroreductase gene (NfsB) into MCS2 using primer pair MZ5_fw/ MZ6_rev between FseI and XhoI restriction sites.

Cloning of pETDuet-1: Enc-Strep-Tag (MCS1); NfsB (MCS2)
The commercially available pETDuet-1 vector (Novagen) was modified to carry a Strep-tag II sequence. Strep-tag II was introduced into MCS1 using an annealed oligonucleotide cloning approach with primer pair MZ7_fw/ MZ8_rev, inserted between SacI and HindIII restriction sites. Subsequently, the open reading frame encoding *msmei_5672* was amplified from the genomic DNA of *M. smegmatis* mc^2155 using primer pair MZ9_fw/ MZ10_rev and inserted into MCS1 between NcoI and SacI restriction sites. Concurrently, MCS2 was modified by introducing the C-terminal ELS sequence between XhoI and PacI restriction sites using primer pair MZ3_fw/ MZ4_rev via annealed oligonucleotide cloning approach. Standard cloning techniques were then employed to insert the *E. coli* nitroreductase gene (NfsB) into MCS2 using primer pair MZ11_fw/ MZ6_rev and NdeI and XhoI restriction enzymes.

To form a tandem dimer, individual NfsB monomers were linked using the NEBuilder HiFi DNA Assembly kit (New England Biolabs, Beverly, MA, USA) with a flexible 22-residue linker sequence LEGSAGQGAQAGQGAQAGSSAG (corresponding DNA sequence: CTCGAGGGATCAGCAGGTCAGGGTGCTCAGGCTGGTCAGGGTGCTCAGGCTGGTAGCTCTGCTGG), generating constructs: pETDuet-1: Strep-tdNfsB (MCS2) and pETDuet-1: Enc-Strep-Tag (MCS1); tdNfsB (MCS2).

Cloning of pETDuet-1: Enc-Strep-Tag (MCS1); eGFP (MCS2)[3]

The commercially available pETDuet-1 vector (Novagen) was modified to carry a Strep-tag II sequence. Strep-tag II was introduced into MCS1 using an annealed oligonucleotide cloning approach with primer pair MZ7_fw/ MZ8_rev, inserted between SacI and HindIII restriction sites. Subsequently, the open reading frame encoding MSMEG_5380 was amplified from the genomic DNA of *M. smegmatis* mc2155 using primer pair MZ9_fw/ MZ10_rev and inserted into MCS1 between NcoI and SacI restriction sites. Concurrently, MCS2 was modified by introducing the C-terminal ELS sequence between XhoI and PacI restriction sites using primer pair MZ3_fw/ MZ4_rev via an annealed oligonucleotide cloning approach. Standard cloning techniques were then employed to insert the eGFP gene into MCS2 using primer pair MZ12_fw/ MZ13_rev and NdeI and XhoI restriction enzymes.

Construction of five-fold pore encapsulin mutants

Five-fold pore encapsulin mutants were generated using the Q5 Site-Directed Mutagenesis Kit (New England Biolabs, Beverly, MA, USA) following the manufacturer's instructions. Specific primer pairs were designed for each mutation and utilized as follows:

For the Δ4-Ala2 mutation, primer pair MZ14_fw and MZ15_rev was employed. The Δ4-Ala1 mutation was generated using primer pair MZ14_fw and MZ16_rev. Primer pair MZ17_fw and MZ18_rev was utilized for the Δ6-Ala3 mutation, while the Δ6-Ala2 mutation was introduced using primer pair MZ19_fw and MZ18_rev.

Table S1. Primers and oligonucleotides used in this study.

Name	Sequence
MZ1_fw	TATGTGGAGCCACCCGCAGTTCGAAAAATGGCCGG
MZ2_rev	CCATTTTTCGAACTGCGGGTGGCTCCACA
MZ3_fw	TCGAGTCACTCGGAATCGGCAGCCTGAAAGGAACCCGCTGATTAAT
MZ4_rev	TAATCAGCGGGTTCCTTTCAGGCTGCCGATTCCGAGTGAC
MZ5_fw	TAAGCAGGCCGGCCGATATCATTTCTGTCGCCTTAAAG
MZ6_rev	GCTTTACTCGAGCACTTCGGTTAAGGTGATGTTTTG
MZ7_fw	CTGGAGCCACCCGCAGTTCGAAAAATGAA
MZ8_rev	AGCTTTCATTTTTCGAACTGCGGGTGGCTCCAGAGCT
MZ9_fw	TAAGCACCATGGGAAACAACCTCTATCGCGACCTCGC
MZ10_rev	TAAGCAGAGCTCGGGGGTCAGCGCGACAGAG
MZ11_fw	TAAGCACATATGGATATCATTTCTGTCGCCTTAAAG
MZ12_fw	GCTTCATATGGTGAGCAAGGGCGAGG
MZ13_rev	GACTCGAGCTTGTACAGCTCGTCCATG
MZ14_fw	GCAATCCGCGAGCACATCAACC
MZ15_rev	CGCTGCGGTGGTCTCGCTGAC
MZ16_rev	TGCGGTGGTCTCGCTGAC
MZ17_fw	GCAGCGATCCGCGAGCACATCAAC
MZ18_rev	CGCGGTCTCGCTGACCTTGGT
MZ19_fw	GCAATCCGCGAGCACATCAAC

Complete protein sequences

Purple: purification tag; Yellow: protein sequence NfsB; Green: protein sequence eGFP; Blue: protein sequence Encapsulin; Brown: linker between monomers; Red: encapsulin localization sequence.

Strep-Tag-NfsB

MWSHPQFEKWPADIISVALKRHSTKAFDASKKLTPEQAEQIKTLLQYSPSSTNSQPWHFIVASTEEGKARVAKSAAGN
YVFNERKMLDASHVVVFCAKTAMDDVWLKLVVDQEDADGRFATPEAKAANDKGRKFFADMHRKDLHDDAEWMAKQVYL
NVGNFLLGVAALGLDAVPIEGFDAAILDAEFGLKEKGYTSLVVVPVGHHSVEDFNATLPKSRLPQNITLTEVLESLGI
GSLKGTR

Strep-Tag-tdNfsB

MWSHPQFEKGGGGSDIISVALKRHSTKAFDASKKLTPEQAEQIKTLLQYSPSSTNSQPWHFIVASTEEGKARVAKSAA
GNYVFNERKMLDASHVVVFCAKTAMDDVWLKLVVDQEDADGRFATPEAKAANDKGRKFFADMHRKDLHDDAEWMAKQV
YLNVGNFLLGVAALGLDAVPIEGFDAAILDAEFGLKEKGYTSLVVVPVGHHSVEDFNATLPKSRLPQNITLTEVLEGS
AGQGAQAGQGAQAGSSAGDIISVALKRHSTKAFDASKKLTPEQAEQIKTLLQYSPSSTNSQPWHFIVASTEEGKARVA
KSAAGNYVFNERKMLDASHVVVFCAKTAMDDVWLKLVVDQEDADGRFATPEAKAANDKGRKFFADMHRKDLHDDAEWM
AKQVYLNVGNFLLGVAALGLDAVPIEGFDAAILDAEFGLKEKGYTSLVVVPVGHHSVEDFNATLPKSRLPQNITLTEV
LESLGIGSLKGTR

Enc-Strep-Tag

MGNNLYRDLAPITESAWAEIELEATRTFKRHIAGRRVVDVSGPNGPTTASVSTGHLLDVSPPGDGVIAHLRDAKPLVR
LRVPFTVARRDIDDVERGSQDSDWDPVKDAAKKLAFVEDRAIFEGYAAASIEGIRSSSSNPALALPDDAREIPDVIAQ
ALSELRLAGVDGPYSVLLSAETYTKVSETTAHGYPIREHINRLVDGEIIWAPAIDGAFVLSTRGGDFDLQLGTDVSIG
YLSHDAEVVHLYMEETMTFLCYTAEASVALTPELWSHPQFEK

NfsB

MDIISVALKRHSTKAFDASKKLTPEQAEQIKTLLQYSPSSTNSQPWHFIVASTEEGKARVAKSAAGNYVFNERKMLDA
SHVVVFCAKTAMDDVWLKLVVDQEDADGRFATPEAKAANDKGRKFFADMHRKDLHDDAEWMAKQVYLNVGNFLLGVAA
LGLDAVPIEGFDAAILDAEFGLKEKGYTSLVVVPVGHHSVEDFNATLPKSRLPQNITLTEVLESLGIGSLKGTR

tdNfsB

MDIISVALKRHSTKAFDASKKLTPEQAEQIKTLLQYSPSSTNSQPWHFIVASTEEGKARVAKSAAGNYVFNERKMLDA
SHVVVFCAKTAMDDVWLKLVVDQEDADGRFATPEAKAANDKGRKFFADMHRKDLHDDAEWMAKQVYLNVGNFLLGVAA
LGLDAVPIEGFDAAILDAEFGLKEKGYTSLVVVPVGHHSVEDFNATLPKSRLPQNITLTEVLEGSAGQGAQAGQGAQA
GSSAGDIISVALKRHSTKAFDASKKLTPEQAEQIKTLLQYSPSSTNSQPWHFIVASTEEGKARVAKSAAGNYVFNERK
MLDASHVVVFCAKTAMDDVWLKLVVDQEDADGRFATPEAKAANDKGRKFFADMHRKDLHDDAEWMAKQVYLNVGNFLL
GVAALGLDAVPIEGFDAAILDAEFGLKEKGYTSLVVVPVGHHSVEDFNATLPKSRLPQNITLTEVLESLGIGSLKGTR

eGFP

MVSKGEELFTGVVPILVELDGDVNGHKFSVSGEGEGDATYGKLTLKFICTTGKLPVPWPTLVTTLTYGVQCFSRYPDH
MKQHDFFKSAMPEGYVQERTIFFKDDGNYKTRAEVKFEGDTLVNRIELKGIDFKEDGNILGHKLEYNYNSHNVYIMAD
KQKNGIKVNFKIRHNIEDGSVQLADHYQQNTPIGDGPVLLPDNHYLSTQSALSKDPNEKRDHMVLLEFVTAAGITLGM
DELYKLESLGIGSLKGTR

Table S2. Molecular structures of compounds applied in this study.

Name	Molecular Weight (Da)	Structure	Reference
AV2	495.5	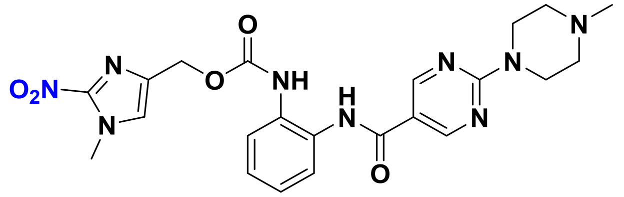	[1]
AV4	495.5	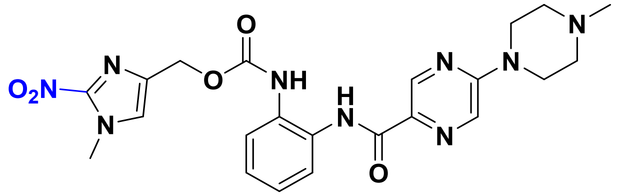	[1]

MA60	452.4	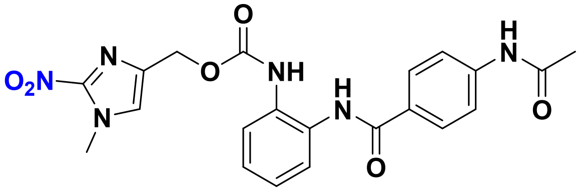	[1]
MA63	534.5	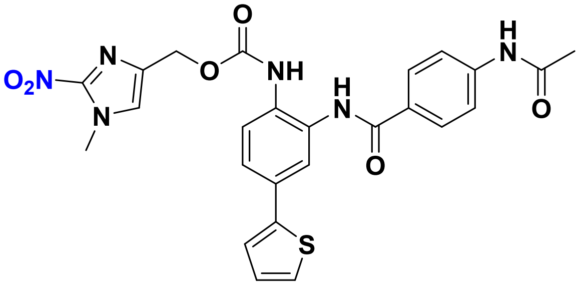	[1]
CB1954	252.2	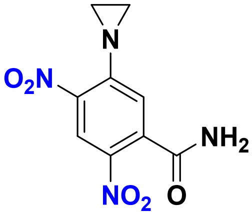	[4,5]
$CoNO_2$	262.3	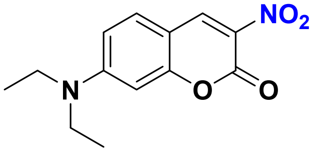	[6]
Nbzp	234.3	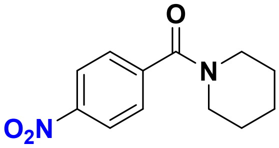	[7]

Table S3. Refinement statistics.

	Open-pentameric pore state	*Closed-pentameric pore state*
Data collection		
Magnification	80,000 x	80,000 x
Voltage (kV)	300	300
Electron exposure e$^-$/Å^2)	60.2	60.2
Defocus range (μm)	-0.5 – -2.0	-0.5 – -2.0
Pixel size (Å)	0.7832	0.7832
Data processing	9FNA, EMD-50586	9FN9, EMD-50585
Symmetry imposed	I	C1
Initial particles	559,602	559,602
Final particles	546,841	308.711
Map resolution (Å)	2.22	2.84
FSC threshold	0.143	0.143
Refinement		
Model resolution (Å)	2.22	2.84
Model composition		
Non-hydrogen atoms	121,020	121,020
Protein residues	15,840	15,840

r.m.s. deviations		
Bond length (Å)	0.003	0.002
Bond angles (˚)	0.686	0.509
Validation		
MolProbability score	1.42	1.16
Clashscore	7.68	3.76
Rotamer outliers (%)	0.47	0.93
CaBLAM outliers (%)	0.77	0
Ramachandran plot (%)		
Outliers	0	0
Allowed	1.14	1.15
Favoured	98.86	98.85

References

1 M. Abdelsalam, M. Zmyslia, K. Schmidtkunz, A. Vecchio, S. Hilscher, H. S. Ibrahim, M. Schutkowski, M. Jung, C. Jessen-Trefzer and W. Sippl, *Archiv der Pharmazie*, 2024, **357**, 2300536.

2 E.-M. Herrlinger, M. Hau, D. M. Redhaber, G. Greve, D. Willmann, S. Steimle, M. Müller, M. Lübbert, C. C. Miething, R. Schüle and M. Jung, *Chembiochem*, 2020, **21**, 2329–2347.

3 P. Lohner, M. Zmyslia, J. Thurn, J. K. Pape, R. Gerasimaitė, J. Keller-Findeisen, S. Groeer, B. Deuringer, R. Süss, A. Walther, S. W. Hell, G. Lukinavičius, T. Hugel and C. Jessen-Trefzer, *Angew Chem Int Ed Engl*, 2021, **60**, 23835–23841.

4 A. Christofferson and J. Wilkie, *Biochem Soc Trans*, 2009, **37**, 413–418.

5 R. J. Knox, F. Friedlos, M. Jarman and J. J. Roberts, *Biochem Pharmacol*, 1988, **37**, 4661–4669.

6 C. Jia, Y. Zhang, Y. Wang and M. Ji, *RSC Adv*, **11**, 8516–8520.

7 T. Güngör, F. C. Önder, E. Tokay, Ü. G. Gülhan, N. Hacıoğlu, T. T. Tok, A. Çelik, F. Köçkar and M. Ay, *Eur. J. Med. Chem.*, 2019, **171**, 383–400.
